# Species-Specific Responses of Juvenile Rockfish to Elevated *p*CO_2_: From Behavior to Genomics

**DOI:** 10.1371/journal.pone.0169670

**Published:** 2017-01-05

**Authors:** Scott L. Hamilton, Cheryl A. Logan, Hamilton W. Fennie, Susan M. Sogard, James P. Barry, April D. Makukhov, Lauren R. Tobosa, Kirsten Boyer, Christopher F. Lovera, Giacomo Bernardi

**Affiliations:** 1 Moss Landing Marine Laboratories, Moss Landing, California, United States of America; 2 California State University Monterey Bay, Seaside, California, United States of America; 3 National Marine Fisheries Service, Southwest Fisheries Science Center, Santa Cruz, California, United States of America; 4 Monterey Bay Aquarium Research Institute, Moss Landing, California, United States of America; 5 Department of Ecology and Evolutionary Biology, University of California Santa Cruz, Santa Cruz, California, United States of America; University of Connecticut, UNITED STATES

## Abstract

In the California Current ecosystem, global climate change is predicted to trigger large-scale changes in ocean chemistry within this century. Ocean acidification—which occurs when increased levels of atmospheric CO_2_ dissolve into the ocean—is one of the biggest potential threats to marine life. In a coastal upwelling system, we compared the effects of chronic exposure to low pH (elevated *p*CO_2_) at four treatment levels (i.e., *p*CO_2_ = ambient [500], moderate [750], high [1900], and extreme [2800 μatm]) on behavior, physiology, and patterns of gene expression in white muscle tissue of juvenile rockfish (genus *Sebastes*), integrating responses from the transcriptome to the whole organism level. Experiments were conducted simultaneously on two closely related species that both inhabit kelp forests, yet differ in early life history traits, to compare high-CO_2_ tolerance among species. Our findings indicate that these congeners express different sensitivities to elevated CO_2_ levels. Copper rockfish (*S*. *caurinus*) exhibited changes in behavioral lateralization, reduced critical swimming speed, depressed aerobic scope, changes in metabolic enzyme activity, and increases in the expression of transcription factors and regulatory genes at high *p*CO_2_ exposure. Blue rockfish (*S*. *mystinus*), in contrast, showed no significant changes in behavior, swimming physiology, or aerobic capacity, but did exhibit significant changes in the expression of muscle structural genes as a function of *p*CO_2_, indicating acclimatization potential. The capacity of long-lived, late to mature, commercially important fish to acclimatize and adapt to changing ocean chemistry over the next 50–100 years is likely dependent on species-specific physiological tolerances.

## Introduction

Global climate change from the burning of fossil fuels is predicted to trigger large-scale changes in ocean chemistry within this century [1,2]. CO_2_ levels have increased dramatically in the ocean over the past two centuries [3], resulting in an average decline in the pH of surface waters by 0.1 units since the industrial revolution. Global ocean pH is predicted to decrease by as much as 0.4 units by 2100 [4], making the ocean more acidic than at any time during the past 400,000 years [1]. Ocean acidification is considered one of the biggest threats to marine life [5,6], although the bulk of past research has primarily focused on calcifying species (coralline algae, corals, molluscs, etc.) [7].

Although teleost fishes have generally been presumed to be tolerant of ocean acidification due to their highly efficient acid-base regulation [8], a recent review concluded that fishes appear to be more sensitive to projected pH changes by the year 2100 than previously thought (72.7% of species responded negatively to elevated *p*CO_2_) [9]. Recent studies of juvenile coral reef fish have indicated that development in low pH water (i.e., high *p*CO_2_) results in the impairment of olfactory senses, such as predator odor cue detection [10], changes in neurologic function (e.g., behavioral lateralization [11]), and limitation of the capacity for aerobic activity [12]. Interestingly, the effects of ocean acidification on anti-predator responses in damselfish indicate that closely related species (4 congeners in the genus *Pomacentrus*) may differ considerably in the sensitivity of behavioral responses to high CO_2_ [13]. Results from studies of temperate fish species have ranged from no observable effects [14], to others demonstrating the occurrence of tissue damage [15], reduced growth and survival [16], and behavioral disruptions after elevated *p*CO_2_ exposure [17,18]. Several of these studies have begun to elucidate the mechanisms underlying responses by fish to elevated *p*CO_2_, e.g., behavioral changes appear to arise from interference with GABA_A_ neurotransmitter function, possibly as a result of changes in Cl^−^ and HCO_3_^−^ ion gradients that occur during acid-base regulation [17, 19].

Environmental hypercapnia, leading to elevated internal *p*CO_2_ levels, may generally alter physiological performance of marine organisms in response to ocean acidification by affecting respiration and overall aerobic capacity, especially for animals with poor ability to compensate for acid/base changes. In fishes, compensation of hypercapnic acidosis occurs within hours to days as HCO_3_^-^ levels in blood plasma increase through net acid secretion or increased HCO_3_^-^ retention/uptake [20]. Although this process buffers pH in extracellular fluids, resultant high *p*CO_2_ and HCO_3_^-^ levels may lead to other downstream effects on behavior, calcification, and osmoregulation [8]. In addition, compensatory changes in acid-base regulation and osmoregulation require energy-intensive ion pumps, and there is the possibility that these energetic costs are large enough to cause an energetic deficit at the level of the whole organism (i.e., leading to shifts in metabolic rate) [8,9,21]. A recent meta-analysis of marine ectotherms, including fishes, indicated that changes in respiratory performance in response to elevated CO_2_ was highly variable across taxa, with some species exhibiting negative impacts, while others exhibited no effect or a positive response [21]. In calcifying species, transcriptomics have been used to evaluate potential changes in energy budgets by uncovering groups of differentially expressed genes involved in ion transport and energy production in response to high *p*CO_2_ [22]. Recently, one of the first studies of transcriptome-wide changes in gene expression in response to elevated *p*CO_2_ and temperature in a fish species reported a strong cellular stress response (metabolic shifts, DNA damage repair, immune system response, etc.) that peaked after 7 days but continued to persist to a limited degree for months following acclimation [23].

It is often assumed that species inhabiting eastern boundary current upwelling systems are less susceptible to ocean acidification than tropical species because they have evolved in an environment characterized by more variable pH (e.g. seasonal upwelling periodically introduces deeper, lower pH [7.5–7.8, depending on location] waters onto the coastal shelf; ref. [1]; S1 Fig). Variability in pH has been observed on a variety of temporal scales in kelp forests and coastal locations throughout California [24,25]. Detailed oceanographic forecasts modeling pH dynamics in the California Current predict that chronic low pH conditions (at least 0.2 pH units lower than current levels) in the upper 60 m of coastal waters (within 10 km from shore) will occur in the next 40 years, on top of the seasonal variability due to upwelling [2]. Thus, many temperate species may already be experiencing end-of-the-century predictions of pH for short durations, but exposure to these low pH conditions is predicted to be more frequent and longer in duration in the future.

We compared the responses of two congeneric rockfish species during chronic exposure (21 weeks) to elevated *p*CO_2_ (i.e., reduced pH). Although both species are common to kelp forests along the U.S. West Coast and have similar adult lifestyles, we hypothesized that differences in early life history traits may influence their tolerance to ocean acidification. Copper rockfish (*Sebastes caurinus*) spawn in spring (February-March), have a ~2 month larval duration, with pelagic stages developing close to the surface, and juveniles settling in the upper water column near the top of the kelp canopy [26,27], where *p*CO_2_ is locally reduced due to kelp photosynthesis (pH can differ by 0.3 units between 7 and 17 m depth in a San Diego kelp bed; ref. [25]). Blue rockfish (*S*. *mystinus*) spawn in winter (December-February), have a 3–4 month pelagic duration, with larvae and pelagic juveniles developing deeper in the water column, and juveniles settling near the benthos [26,27], where *p*CO_2_ is locally elevated due to respiration of benthic organisms and intrusion of upwelling plumes (see ref. [25] and S1 Fig for conditions at our study site). Thus, blue rockfish are typically exposed to higher *p*CO_2_ levels during development and are predicted to be better adapted to future conditions compared to copper rockfish. However, increased sensitivity of copper rockfish could also be a result of their smaller size and younger age at settlement. We collected juveniles of both species at comparable post-settlement development stages (1–2 weeks post-settlement) from kelp beds in central California and reared them in the laboratory (*see* Methods) at four different *p*CO_2_ levels (Table 1), reflecting conditions predicted to occur in the next 50–100 years and beyond [2,28]. After set exposure durations (Table 2), individual fish were run successively through a series of behavioral and physiological challenges and then sacrificed for transcriptomic assays of gene expression using RNA sequencing.

**Table 1:** Mean carbonate chemistry conditions (± standard error) in the experimental system. Shown are mean values of dissolved organic carbon (DIC), total alkalinity, pH (total scale), *p*CO_2_, and temperature.

Treatment	pH	*p*CO_2_ (μatm)	DIC (μmol/kg)	Total Alkalinity (μmol/kg)	Temperature (°C)
Ambient	7.87 (0.01)	546.3 (22.3)	2101.8 (3.9)	2223.0 (5.9)	10.82 (0.29)
Moderate	7.74 (0.04)	749.0 (5.2)	2157.3 (6.9)	2223.1 (6.5)	10.62 (0.04)
High	7.49 (0.01)	1898.9 (68.5)	2259.2 (4.8)	2223.3 (4.8)	10.58 (0.13)
Extreme	7.32 (0.02)	2803.6 (363.4)	2338.0 (5.8)	2236.1 (17.1)	10.70 (0.04)

**Table 2:** Summary of the range of exposure duration, acclimation time, time per trial, recovery period, and sample size for copper and blue rockfish used to test behavioral and physiological responses to elevated *p*CO_2_. Note: Individual fish were used successively in the different trials to enable tracking of performance measures. Data from fish that did not behave normally in a particular trial were excluded (e.g., refusal to swim in the U_crit_ test). In addition, 2 of 12 blue rockfish individuals that were sequenced had low quality reads and were subsequently excluded from the differential gene expression analysis.

	U_crit_	Lateralization	Aerobic scope	Transcriptomics
**A. Copper rockfish**				
Cumulative *p*CO_2_ exposure	5–8 weeks	10 weeks	14–17 weeks	21 weeks
Acclimation time after handling	15 min	3 min	2 hrs	NA
Experimental duration	15 min	15 min	1.5 hrs	NA
Recovery period until next trial	7–21 days	7–14 days	3–6 weeks	NA
Samples size (*n*)	*n* = 25	*n* = 29	*n* = 29	*n* = 15
				
**B. Blue rockfish**				
Cumulative *p*CO_2_ exposure	7–9 weeks	10 weeks	16–19 weeks	21 weeks
Acclimation time after handling	15 min	3 min	2 hrs	NA
Total experimental duration	15 min	15 min	1.5 hrs	NA
Recovery period until next trial	7–21 days	7–14 days	1–4 weeks	NA
Sample size (*n*)	*n* = 30	*n* = 33	*n* = 34	*n* = 10

## Results and Discussion

### Behavioral and physiological responses to elevated *p*CO_2_

**Fig 1:**
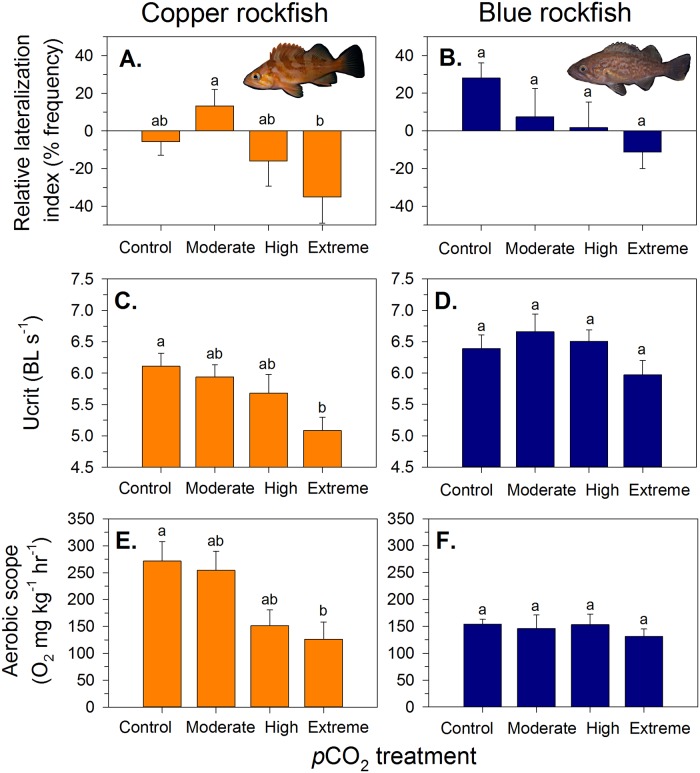
Changes in behavioral lateralization, critical swimming speed, and aerobic scope of juvenile copper and blue rockfish as a function of *p*CO_2_ treatment exposure history. (A, B) Behavioral lateralization is measured using the relative lateralization index (negative values = right turn bias in a detour test). (C,D) Critical swimming speed (U_crit_) is the maximum sustained speed in body lengths per second. (E,F) Aerobic scope represents the difference between maximum and resting metabolic rates (measured as oxygen consumption) and is a proxy for the capacity for aerobic activity. Bars are mean values (± SE). Letters over bars represent results of Tukey HSD post-hoc tests; significantly different means do not share letters in common. Note: Due to logistical constraints all behavioral and physiological trials occurred in control seawater (*p*CO_2_ ~550 μatms).

Behavioral lateralization is a test of brain functional asymmetry and is commonly used as a proxy to examine changes in neural processing in response to stimuli [11]. The degree of individual lateralization (bias for left vs. right turning decisions) can affect performance in cognitive tasks, schooling behavior, spatial orientation, and escape reactions from predators. Copper rockfish exhibited a significant shift in the relative lateralization index (ANOVA, *F*_*3*,*25*_ = 3.82, *P* = 0.022; Fig 1A), becoming more right turn biased in detour tests between the moderate (750 μatm) and extreme (2800 μatm) *p*CO_2_ treatments. In contrast, no significant lateralization bias was detected in blue rockfish (ANOVA, *F*_*3*,*29*_ = 1.23, *P* = 0.32; Fig 1B), despite a trend for a shift from left to right turn bias in detour tests at higher *p*CO_2_ levels. Statistical power, however, was relatively low for this analysis (β = 0.3) due to the high variability among fish and a sample size of *n* = 73 would be required to detect a difference at the effect size measured. The absolute lateralization index (whether fish are lateralized regardless of turning direction) indicated no significant differences in either species (copper: ANOVA, *F*_*3*,*28*_ = 2.10, *P* = 0.125; blue: ANOVA *F*_*3*,*29*_ = 0.83, *P* = 0.49) across the *p*CO_2_ treatments (S2 Fig). However, copper rockfish did show a trend to becoming more lateralized at higher *p*CO_2_ exposures. Tropical damselfish [11,18] and temperate sticklebacks [17] have been reported to exhibit significant shifts in behavioral lateralization with increasing exposure to high *p*CO_2_. Those species were typically highly lateralized at low *p*CO_2_ and became less lateralized at high *p*CO_2_ (in the range of 1000 μatm). Copper rockfish exhibited the opposite pattern, becoming more lateralized and shifting from a right to a slightly left turn bias at the extreme CO_2_ levels, whereas we could not detect a significant effect of elevated *p*CO_2_ on blue rockfish. It remains unclear how the increased lateralization in rockfishes may impact performance or subsequent fitness.

Aerobic critical swimming speed (U_crit_), tested in a swim tunnel, is similar to an exercise stress test in that it measures the maximum relative swimming velocity (in body lengths per second) a fish can sustain before fatiguing [29,30]. For copper rockfish, U_crit_ declined significantly as a function of increasing *p*CO_2_ (ANOVA, *F*_*3*,*21*_ = 3.79, *P* = 0.026; Fig 1C), such that fish in the extreme *p*CO_2_ treatment exhibited a 16.8% decline in critical swimming speed compared to the control group. In contrast, blue rockfish did not exhibit significant variation in U_crit_ (ANOVA, *F*_*3*,*26*_ = 1.71, *P* = 0.19; Fig 1D) across the *p*CO_2_ treatments. Differences in U_crit_ among treatments were not related to growth rate for either species; growth did not differ among treatments for copper rockfish (ANOVA, *F*_*3*,*26*_ = 1.61, *P* = 0.21), but was reduced in the high *p*CO_2_ treatment for blue rockfish (ANOVA, *F*_*3*,*31*_ = 4.55, *P* = 0.01) (S3 Fig). Declines in critical swimming speed may influence the predator escape response, and U_crit_ has been found to correlate with other ecologically relevant responses, such as routine activity levels, metabolic rate, and body size [29]. Blue rockfish are fairly active swimmers in the water column, whereas copper rockfish are more sedentary and only swim actively when chased by a predator, which may explain the heightened sensitivity to high CO_2_ exposure in copper rockfish during the U_crit_ trial. This represents one of the first studies to report a significant change in U_crit_ in response to elevated *p*CO_2_, although other studies have examined this response and found no effect [31–33].

Aerobic scope measures an organism’s capacity for aerobic activity and may be a proxy for whole organismal performance and fitness [34]. It is calculated as the difference between standard (or routine) metabolic rate and maximum metabolic rate (e.g., when swimming near the maximum sustained velocity) [12]. Copper rockfish displayed a significant depression in aerobic scope (ANOVA; *F*_*3*,*28*_ = 4.538, *P* = 0.0103; Fig 1E) by 53.5% in the highest *p*CO_2_ treatments compared to the control. The decline in aerobic scope was driven by a marginally non-significant decline of maximum oxygen consumption rates in the high and extreme high *p*CO_2_ treatments (ANOVA, *F*_*3*,*28*_ = 2.574, *P* = 0.074) and no significant difference in routine oxygen uptake rates (ANOVA, *F*_*3*,*28*_ = 0.850, *P* = 0.479) across the *p*CO_2_ treatments (S4 Fig). In contrast to copper rockfish, blue rockfish did not change in aerobic scope (ANOVA, *F*_*3*,*30*_ = 0.448, *P* = 0.728; Fig 1F) across the four *p*CO_2_ treatments. Neither resting oxygen consumption (ANOVA, *F*_*3*,*30*_ = 1.947, *P* = 0.143) nor maximum oxygen consumption rate (ANOVA, *F*_*3*,*30*_ = 2.253, *P* = 0.103) differed as a function of *p*CO_2_ exposure history for blue rockfish (S4 Fig).

Reduced aerobic scope and swimming capability (U_crit_), driven by a decrease in maximum metabolic rate (MMR), could be a result of “limiting stress” (defined in ref. [35]). It has been hypothesized that elevated *p*CO_2_ could reduce oxygen uptake from the environment due to acidification of blood and respiratory pigments [36]. Although we expect that both species had compensated blood plasma pH after chronic exposure to elevated *p*CO_2_ (similar to ref. [20]), we did not measure blood chemistry in our experiments. A decrease in aerobic scope mediated by a decrease in MMR under high *p*CO_2_ has been observed in tropical cardinalfish [12]. In contrast, two other studies showed the opposite effect, with MMR increasing under high *p*CO_2_ [37,38], potentially in response to other effects on respiratory physiology or behavior. These studies and a recent meta-analysis [21] highlight the taxonomic differences in the tolerances of teleost fishes to high *p*CO_2_. On average, exposure to high CO_2_ results in a depression of aerobic scope, with little detectable impact on resting metabolic rate [21], similar to the trends we reported, although the results are highly variable across fish species.

Copper rockfish had approximately twice the mass-specific routine and maximum metabolic rate as blue rockfish (S4 Fig), which could be explained by their relative differences in size (average weight for coppers: 1.60 g ± 0.67 SE; blues: 3.23 g ± 0.94 SE), morphology, or behavior. Blue rockfish have higher baseline activity levels in the kelp forest (i.e., recently settled blue rockfish are more active swimmers in the midwater, while copper rockfish shelter near kelp blades) and their body shape is more elongate and streamlined. The higher aerobic scope and significant decline in this trait observed for copper rockfish may reflect the heightened sensitivity of this species to elevated *p*CO_2_, given the greater need to supply oxygen to support metabolic demands during periods of high activity levels and acute physiological stress.

**Fig 2:**
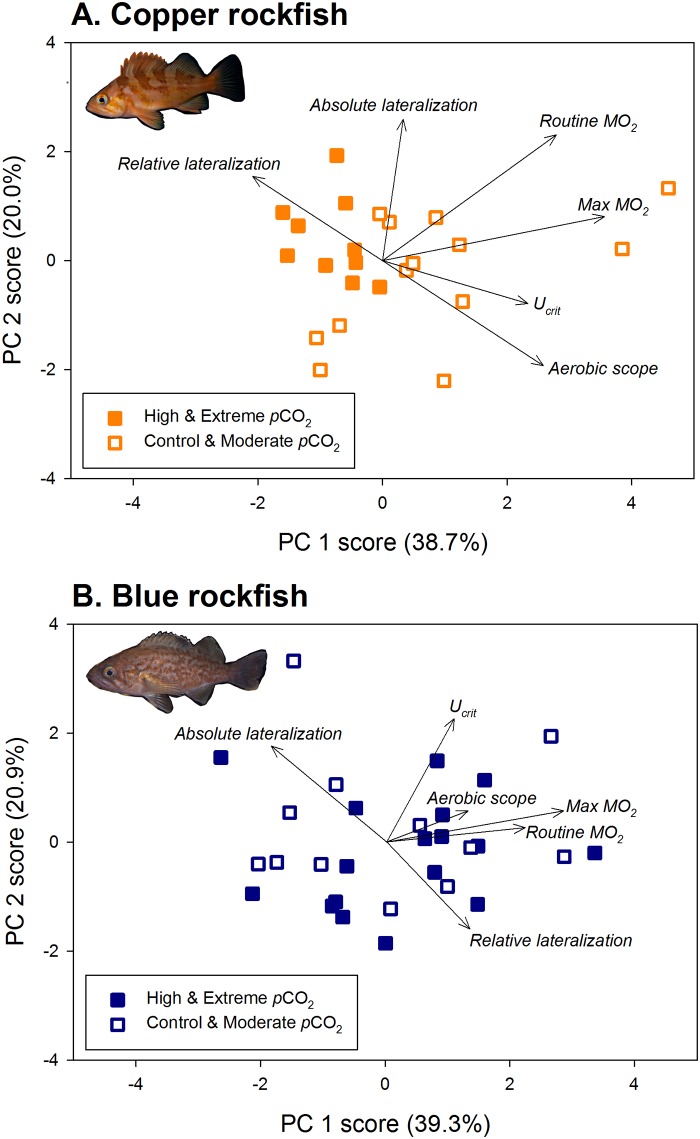
Principal components analysis depicting correlations amongst the suite of behavioral and physiological performance variables measured for (A) copper rockfish and (B) blue rockfish throughout the course of the experiment as a function of *p*CO_2_ exposure history. Filled symbols indicate fish from the two highest *p*CO_2_ treatments and open symbols signify fish from the two lowest *p*CO_2_ treatments. Fish were tagged to allow tracking of individuals across all performance measures and are plotted based on their multivariate combined performance history in behavioral and physiological challenges. Axes show the percent of variation explained by each principal component.

Each individual fish was marked with a unique subcutaneous tag to permit tracking of performance measures as a function of *p*CO_2_ treatment among the experimental trials over the course of the study. To examine correlations among the performance measures, we conducted a principal components analysis for each species, grouping fish into the lowest (control and moderate) and highest (high and extreme) *p*CO_2_ treatments (Fig 2). For copper rockfish, fish from the high and low CO_2_ groups fell into distinct clusters mainly separated along the PC 1 axis (explaining 38.7% of the variation). Fish from high CO_2_ treatments exhibited high scores for relative lateralization, but low values for aerobic scope, U_crit_, and max MO_2_. Thus, individuals that were poor swimmers also displayed reduced aerobic capacity and impaired behavior. Blue rockfish, in contrast, showed no clustering along either PC axis as a function of their *p*CO_2_ exposure history (Fig 2). However, like copper rockfish, there was evidence that individuals with a high aerobic scope and respiration rates were also strong swimmers in the trials. By using a longitudinal study and analyzing the suite of traits measured, our results demonstrate that copper and blue rockfish respond differently to chronic exposure to high CO_2_ and that individuals that ranked highly in performance in one trial subsequently performed well in other trials, while those that performed poorly in one type of trial, continued to do so in other trials.

Similar behavioral and physiological impairments in response to elevated *p*CO_2_ have been reported for other fish species (reviewed in refs. [8,9,21]). Juvenile splitnose rockfish (*S*. *diploproa*) displayed increased anxiety levels following exposure to elevated *p*CO_2_ (*p*CO_2_ = 1125 μatm, pH = 7.75) [17]. This behavioral disruption was stimulated in control fish upon administration of a GABA_A_ receptor antagonist, indicating that elevated CO_2_ alters behavior by impairing neurotransmitter function in the brain, comparable to the effects reported for changes in damselfish lateralization and olfaction [19]. Exposure to very extreme *p*CO_2_ (pH = 7.3, *p*CO_2_ = 5000 μatm) over a 10-day period in the gilt-head bream has been shown to increase reliance on anaerobic metabolism in muscle and cardiac tissues [39]. Studies of the effects of ocean acidification on temperate fish have returned conflicting results. In Atlantic herring, no effects of elevated *p*CO_2_ on embryo development were detected [14], even at extreme levels (pH = 7.05, *p*CO_2_ = 4600). However, exposure to extreme *p*CO_2_ (up to 4200 μatm) resulted in severe tissue damage in Atlantic cod larvae [15] and decreased egg survival, reduced larval size at hatching, and increased occurrence of deformities in inland silversides [16]. Behavioral disruptions in sticklebacks were also observed following exposure to elevated *p*CO_2_ (~1000 μatm, pH = 7.6), with increased impairment after longer exposures [18]. Few studies have tested how multiple related fish species respond to high CO_2_, similar to our tests on copper and blue rockfish. In one study, comparisons of changes in anti-predator behavior (response to injured conspecific cues) among 4 congener damselfish indicated that even closely related species are highly variable in their tolerance to elevated CO_2_ [13]. The four species of *Pomacentrus* tested shared the same ecology, life history, and habitat on coral reefs, yet the observed CO_2_-induced loss of the predation risk response ranged from 30% to 95% among species. In another study that examined the effects of multiple stressors (ocean acidification and hypoxia) on the early life history of three species of estuarine fish, the researchers reported similarly that the three species, two from the same genus (*Menidia*), exhibited dramatic differences in their sensitivity [40]. Thus, the emerging evidence highlights the risk of assuming that even closely related species will respond similarly and the need to test physiological tolerances to high CO_2_ more broadly.

Due to logistical constraints, water used in the larger tanks and flumes for behavioral and physiological challenges consisted of seawater pulled from the Monterey Canyon (i.e., control *p*CO_2_ water). Thus, fish from the elevated *p*CO_2_ treatments experienced a short-term change in *p*CO_2_ during challenges (lasting 20 min to 3 hrs depending on the experiment; Table 2). The change in CO_2_ tension may have resulted in acute alkalosis, which could affect multiple physiological systems. In particular, blood pH and plasma *p*CO_2_ can change relatively quickly (30 min to 1 day) whereas plasma HCO_3_^-^ levels take longer to equilibrate (8 hours to 5 days) following transfer to a new *p*CO_2_ level [20,39]. In contrast, intracellular pH of muscle tissue (which would affect swimming physiology) responds to changes in *p*CO_2_ on an even slower time scale (approximately 1–3 days, ref. [20,39]). An acute change in blood pH could affect oxygen transport and hemoglobin-O_2_ binding. Increases in blood pH (alkalosis) and stable muscle tissue pH (which our fish likely experienced) are expected to result in increased blood oxygen affinity, which would potentially increase oxygen uptake from the environment (loading), with little change in the ability to deliver oxygen to the tissues (unloading). During this short timeframe, respiratory compensation and potentially metabolic compensation may have begun. The effect of short-term alkalosis would have been greatest for fish transferred from the highest *p*CO_2_ treatments and in this scenario would be expected to enhance respiratory performance, which is the opposite of what we observed. Nevertheless, we cannot rule out the possibility that acute alkalosis contributed to the physiological and behavioral responses that we measured.

Studies have shown little effect of transferring fish to a different *p*CO_2_ level on the swimming capabilities of larval clownfish [31] or larval cobia [33] following an hour of acclimation, and little impact of elevated *p*CO_2_ on critical swimming speed at the larval stage, even at *p*CO_2_ levels up to 2100 μatm [33]. In addition, previous research on larval and juvenile damselfish indicated that behavioral impairment (lateralization and olfaction) occurs after several days of exposure to high CO_2_ and impairment is retained for several days after larvae are returned to low CO_2_ conditions [10,19]. Similarly, previous studies on juvenile rockfish indicated that exposure to high CO_2_ conditions increased anxiety and that anxiety levels remained elevated for over 7 days after returning the fish to control CO_2_ conditions [17]. While the effects of ocean acidification on behaviors appear to be reversible, behavioral impairments often persist for days. Thus, fluctuations in pH over the shorter-term (i.e. 30 min to 3 hrs), as experienced by our test subjects, do not seem to mediate the behavioral or physiological effects of high CO_2_ in acclimated individuals. Regardless of whether short-term alkalosis affected behavioral or physiological responses, the two species of juvenile rockfish tested here consistently responded differently to high CO_2_ exposure during the experimental trials conducted in ambient CO_2_ water, and they were handled in the same way, indicating that *p*CO_2_ effects on behavior and physiology were expressed differently in these congeners.

### Gene expression and enzyme activity changes in response to elevated *p*CO_2_

**Fig 3:**
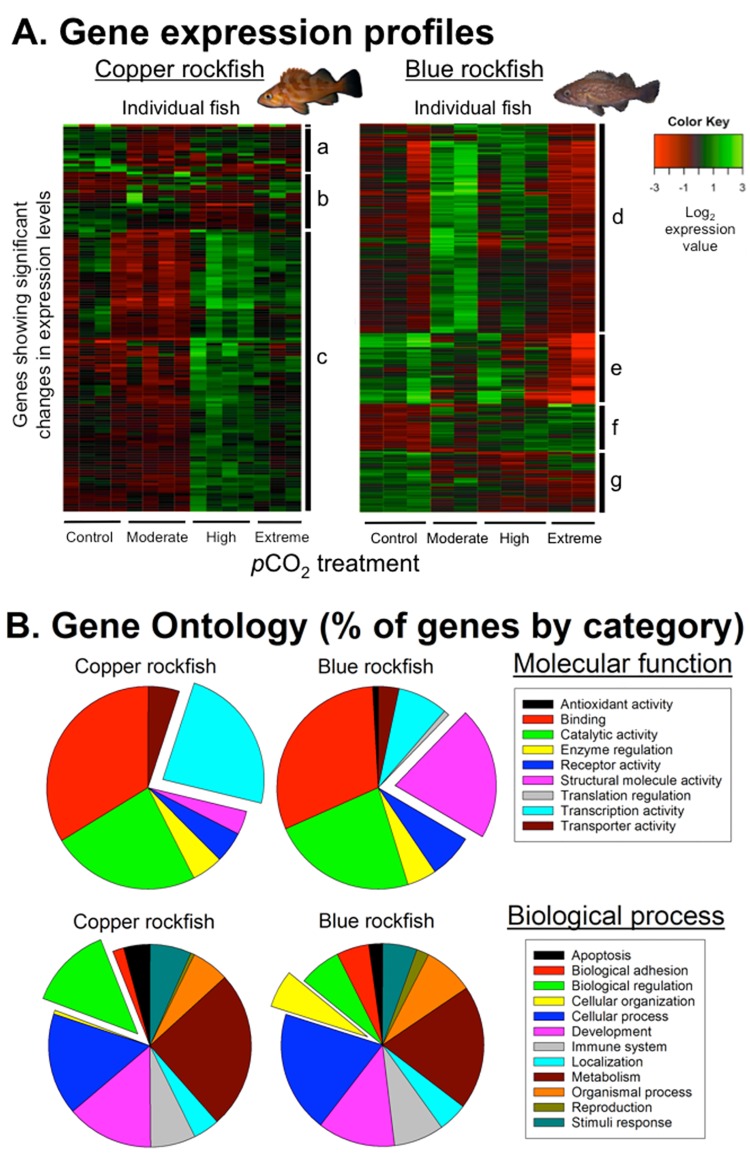
Gene expression profiles (A) and Gene Ontology (GO) functional categories (B) for copper and blue rockfish muscle tissue as a function of *p*CO_2_ treatment. (A) Heatmaps display significant differential gene expression (DE) for copper (*n* = 147) and blue (*n* = 358) rockfish (FDR<0.001) among *p*CO_2_ treatments; green = up-regulation, red = down-regulation. Each column represents an individual fish (*n* = 15 copper rockfish and *n* = 10 blue rockfish). Genes are ordered by similarity in gene expression profile and differ in both order and identity between the two species (only 14 DE genes were in common between the two species). Hierarchical (Euclidean) clustering was used to group similar gene expression profiles, labeled along the right side of each heatmap and listed in S2 and S3 Tables. (B) GO categories show relative differences between copper and blue rockfish in the percentage of annotated genes that were differentially expressed, as classified by GO molecular function or biological process. Broken out pie wedges highlight GO categories that were more expressed in one species than the other. Copper rockfish show significant up-regulation of genes involved in transcription and biological regulation at high *p*CO_2_ and down-regulation at low *p*CO_2_. In contrast, blue rockfish differentially express muscle structural genes across *p*CO_2_ treatments.

RNA sequencing is a powerful next generation sequencing technique that can be used to examine the differential expression of 100s to 1000s of genes in response to environmental stress [41]. We used transcriptomics to generate hypotheses about molecular and biochemical changes underlying observed physiological phenotypes. Phenotypes ultimately arise from changes in gene expression and gene complexes, but changes in mRNA level do not always correspond to a direct change in protein level or enzyme activity. Thus, extrapolation of gene expression results to changes that may be occurring at higher levels of biological organization should be interpreted as hypotheses that require further investigation. Based on the swimming physiology results, we selected white muscle tissue from the dorsal region and used Illumina RNAseq to create a high quality *de novo* transcriptome assembly for copper rockfish (*see* Methods; N50 = 2,536; S1 Table). We then examined the effects of *p*CO_2_ exposure on the transcriptomes for both congeners. We performed an unsupervised PCA on all genes to compare relationships among sample replicates (S5 Fig), and found that the relationships among treatment groups were similar to the patterns observed among differentially expressed genes (Fig 3).

Differential expression analysis showed that copper rockfish exhibited significant differences in expression among *p*CO_2_ treatments (p-value cutoff for false discovery rate [FDR] of <0.001) for 147 genes (Fig 3A), most of which were significantly down-regulated in the low *p*CO_2_ treatments (500 and 800 μatm), and up-regulated in the high *p*CO_2_ treatments (2000 and 3200 μatm), the point at which behavioral and physiological impairment was greatest (Fig 1). The highest number of genes (104 of 147) differentially expressed (DE) between any two treatments occurred between the two mid-range *p*CO_2_ levels (750 and 1900 μatm), shown in “cluster c” on the heatmap (Fig 3A, left). The genes that make up this cluster encode proteins involved in transcription (24/61 = 39%), signaling (10/61 = 16%), and cellular stress response (8/61 = 13%) (S2 Table). While it is difficult to assess the specific biological processes regulated by these transcription factors and signaling molecules, this pattern indicates that there is strong differential regulation occurring between the two highest and two lowest *p*CO_2_ treatments (Fig 3, S5 Fig). Cellular stress response (CSR) genes encode proteins involved in molecular chaperoning (HSP70), response to DNA damage (GADD45), oxidative stress (NOXO1, DUSP), ubiquitination (UBE2E1, UBE2H), apoptosis (BCL2L14, NFKBIA), and transcription factors (C/EBPD, C/EBPB) known to be responsive to thermal and hypoxia stress. Together, these genes indicate a potential sustained cellular stress response, or a cellular homeostatic response (CHR) (*sensu* ref. [38]), after chronic exposure to 1900 μatm *p*CO_2_ and above in copper rockfish. Maintaining a sustained stress response could result in higher overall maintenance costs [39]. This is one potential mechanism that could explain the decline in aerobic scope and critical swimming speed observed in copper rockfish exposed to elevated CO_2_.

In contrast to copper rockfish, blue rockfish exhibited significant differences (FDR <0.001) in the expression profiles of 358 genes (Fig 3A), with up-regulation highest at *p*CO_2_ of 750 and 1900 μatm, and lowest in the extreme *p*CO_2_ treatment (Fig 3A). The highest number of significant DE genes for any pairwise comparison (173 of 358) was between the extreme *p*CO_2_ treatment (2800 μatm) and the moderate treatment (750 μatm), illustrated by “cluster d” on the heatmap (Fig 3A, right). This cluster is dominated by genes encoding proteins involved in muscle contraction (32/126 = 25%), with strong down-regulation in myosins, tropomyosins, troponins, and contractile regulatory genes (e.g., SR Ca^+2^ ATPase, ryanodine receptor, and calsequestrin) at 2800 μatm (S3 Table). The high percentage of muscle contractile genes differentially expressed in blue rockfish at 2800 μatm could be indicative of structural remodeling of muscle tissue, a result potentially consistent with our observation that U_crit_ and aerobic scope were not significantly reduced at higher *p*CO_2_ treatments in this species. For example, tissue remodeling could be a plastic response that enables blue rockfish to compensate against the effects of acidosis. Alternatively, these expression changes could represent down-regulation of isoforms that do not function optimally under high *p*CO_2_. Other prominent functional categories within “cluster d” include signaling (12/126 = 10%), metabolism (11/126 = 9%), cellular structure (10/126 = 8%), and transcription (7/126 = 6%) (S3 Table). In addition, seven DE genes were stress response genes encoding proteins involved in molecular chaperoning (HSP70, HSPB11, HSPB7), oxidative stress (UCP2, superoxide dismutase, eosinophil peroxidase), DNA damage (GADD45), as well as C/EBPD (S3 Table). Interestingly, these stress response genes show an expression profile opposite to that seen in copper rockfish, with highest up-regulation in the mid-range treatments (750 and 1900 uatm), and relative down-regulation in the extreme *p*CO_2_ treatment (2800 μatm). Overall, it appears that there is a threshold above 2000 μatm that results in a major gene regulatory shift in blue rockfish. These DE changes could be an indication that 2800 μatm is causing sublethal stress not apparent at the physiological level, or could represent an additional suite of genes enabling fish to acclimate to the extreme high *p*CO_2_ exposure.

Although the vast majority of significant DE genes (96%) were unique to each species (only 14 unique genes were common to both species and none followed a similar expression profile across treatments) (S2 and S3 Tables), broad gene ontology (GO) categories were similar (Fig 3B). Nevertheless, we found that certain GO categories were enriched in copper versus blue rockfish (Fig 3B) compared to the full transcriptome assembly (S6 Fig), further supporting some of the observations made based on each species’ DE gene list. In copper rockfish, for example, 23.8% of genes were involved in “transcription factor activity” and 13.3% in “biological regulation”, as compared with 8.0% and 6.7%, respectively, in blue rockfish (Fig 3B). The higher percent of genes encoding transcription factors and regulatory proteins suggests larger shifts in regulatory pathways of copper rockfish in response to elevated CO_2_ exposure. In contrast, for blue rockfish 21.2% of genes were involved in “structural molecule activity” and 6% in “cellular component organization” (Fig 3B), versus 3.8% and 0.6%, respectively, in copper rockfish. As discussed previously, these genes were primarily comprised of muscle contractile machinery and were down-regulated in the highest *p*CO_2_ treatment.

To our knowledge, this is the first study that examines fish transcriptomes in combination with changes in whole organismal physiology following exposure to chronic elevation in *p*CO_2_. Our results clearly demonstrate divergent molecular responses between the more tolerant (blue rockfish) and the more susceptible (copper rockfish) congeners. Thus, our study also provides an important baseline to which future ocean acidification studies can be compared. The power of our experimental approach is that we were able to measure behavioral, physiological, and transcriptomic changes in the same individual. It is important to note that extensive acute changes in gene expression likely occurred during the first few weeks of exposure. For example, rapid changes in gene expression occurred in the first 7 days (~2500 DE genes) of exposure to high CO_2_ in the Antarctic fish *Trematous bernacchii*, but then returned closer to normal levels after 56 days (420 DE genes) [23]. Thus, the gene expression profiles we measured in juvenile rockfish after several months are representative of long-term acclimation, or, the case of the high *p*CO_2_ treatments, a state of chronic stress.

**Fig 4:**
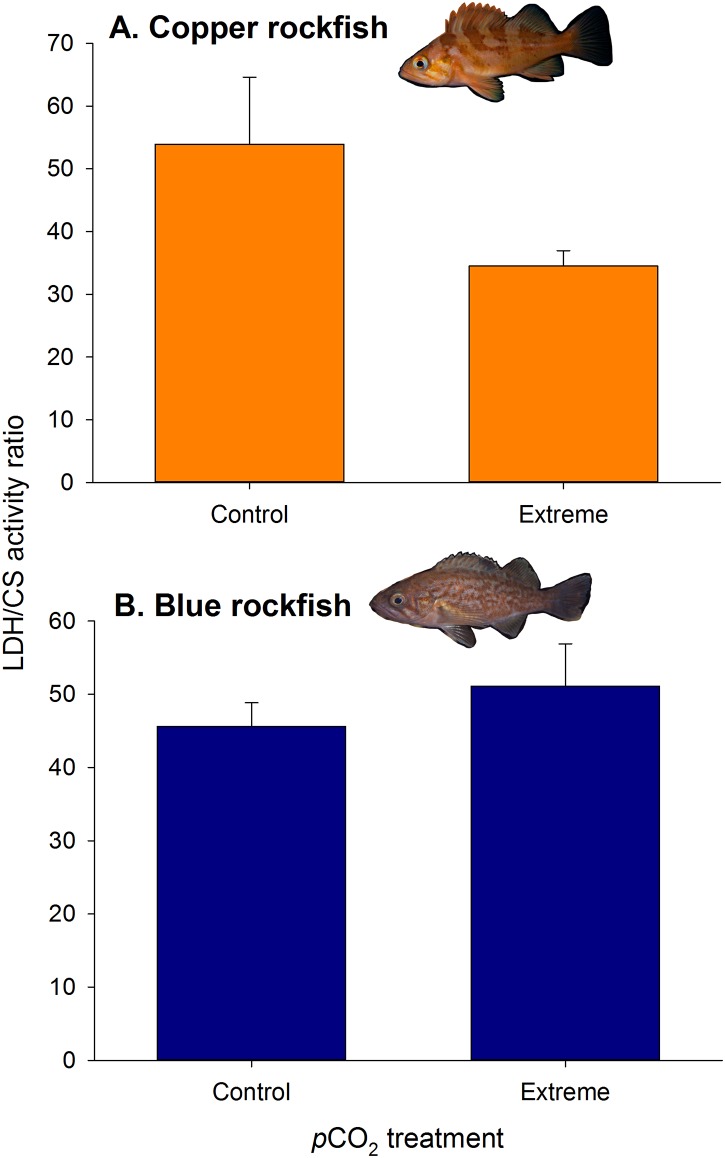
Enzyme activity ratios for lactate dehydrogenase and citrate synthase (LDH:CS) in white muscle tissue of (A) copper and (B) blue rockfish following chronic exposure to extreme *p*CO_2_ (~2800 μatms) or control (~550 μatms) treatments. Copper rockfish exhibited a significant increase in aerobic enzyme activity relative to anaerobic activity (i.e., lower LDH:CS ratio) from the control to extreme high *p*CO_2_ treatments (two-way ANOVA; Species×Treatment: *F*_*1*,*24*_ = 4.46, *P* = 0.045), whereas blue rockfish did not differ in LDH:CS activity among treatments.

To examine whether the physiological and gene expression responses were reflective of shifts in aerobic to anaerobic metabolism at the biochemical level, we measured activity levels of citrate synthase (aerobic) and lactate dehydrogenase (anaerobic) enzymes in muscle tissue. Copper rockfish exhibited a significant increase in aerobic enzyme activity relative to anaerobic activity (i.e., lower LDH:CS ratio) from the control to extreme high *p*CO_2_ treatments, whereas blue rockfish did not differ in LDH:CS activity among treatments (two-way ANOVA; Species×Treatment: *F*_*1*,*24*_ = 4.46, *P* = 0.045) (Fig 4); this inconsistency in the response among species obscured species or treatment effects (two-way ANOVA; Species: *F*_*1*,*24*_ = 0.49, *P* = 0.49; Treatment: *F*_*1*,*24*_ = 1.39, *P* = 0.25). This contrast suggests that copper rockfish had an increased tissue oxygen demand at rest in the extreme *p*CO_2_ treatment that was not exhibited by blue rockfish, although such a demand was not reflected in the whole organismal level routine metabolic rates (S4 Fig). Interestingly, there was not a concomitant increase in expression of genes involved in aerobic metabolism (Fig 3, S3 Table) in copper rockfish as would be expected given the relative increase in aerobic enzyme activity that was observed under the extreme *p*CO_2_ treatment. Overall, our results provide evidence that muscle energy utilization capacity may be altered in the extreme *p*CO_2_ treatment in copper rockfish, and that blue rockfish were able to operate optimally with less oxygen than coppers under high *p*CO_2_ stress.

### Conclusions

Rockfish and other groundfish species of the California Current ecosystem are the backbone of west coast commercial and recreational fisheries. Juvenile rockfish are key prey items for seabirds, marine mammals, salmon and many other higher trophic level predators. Since they have not yet acquired full physiological capacity, early life history stages are predicted to be more sensitive to shifts in ocean chemistry. In turn, early life stages are most critical to population replenishment due to the importance of small changes in early survival in modifying recruitment success. Thus, exposure of early life stages to low pH water may have a disproportionate effect on population dynamics by impairing performance during critical periods of development. We found that recently settled juveniles of two congeneric rockfish differ in their sensitivity to elevated *p*CO_2_, reflected in species-specific behavioral, physiological, biochemical, and genomic responses following extended exposure to four *p*CO_2_ levels in laboratory aquaria. Copper rockfish displayed shifts in behavioral lateralization, depression of critical swimming speed and aerobic scope, relative increases in aerobic enzyme activity at the highest *p*CO_2_ treatment (2800 μatm), and increased relative expression of transcription factors and regulatory genes in the two highest *p*CO_2_ treatments (1900–2800 μatm). While these *p*CO_2_ levels appear extreme relative to the global average of surface waters, similarly low pH conditions already occur naturally for short periods in upwelling zones, like our study system on the central California coast (S1 Fig), and these high CO_2_ events are predicted to increase in magnitude and duration in the near future, potentially reaching chronic high levels that persist for extended durations in the next 100 years [1,2]. In contrast to copper rockfish, blue rockfish exhibited little behavioral or physiological impairment to simulated future *p*CO_2_ conditions, but did differentially express genes involved in muscle structure and function. These species-specific differences may be explained by acclimatization or genetic adaptation to different histories of *p*CO_2_ exposure experienced during early life stages [42,43]. For example, these congeners differ in the seasonal timing of spawning, duration of the pelagic phase, and habitats or depths occupied by early life stages. Likewise, these differences could be a result of differences in size and/or age between the two species at settlement. Further study is needed to disentangle the mechanisms driving differences in the sensitivity of closely related species to future changes in ocean chemistry.

## Materials and Methods

### Ethics statement

The Institutional Animal Care and Use Committee at San Jose State University approved this research on protocols #986 and #1007. Scientific collecting to hold specimens in captivity was permitted by the California Department of Fish and Wildlife on permit #SC-12250.

### Fish collections

Field collections of recently settled copper (30–40 mm total length and <1g) and blue (40–50 mm total length and ~1.5g) rockfish occurred during mid-May to early-June 2013 at Stillwater Cove, Carmel Bay, California (36°33.656’N, 121°56.759’W) from shallow (10–20 m depth) rocky reefs in the center of a large kelp bed. The juveniles of each species were different ages and sizes upon collection due to differing lengths of time spent during the pelagic stage (3–4 months for blues and 2 months for coppers). However, we targeted these sizes specifically for comparison to represent the same post-settlement developmental stage, in terms of length of time spent in the kelp forest after transitioning from the plankton (collections occurred ~1–2 weeks post-settlement). At the end of the experiments size differences in length (coppers: 51.9 mm ± 0.68 SE; blues: 64.6 mm ± 0.53 SE) and weight (coppers: 1.60 g ± 0.67 SE; blues: 3.23 g ± 0.94 SE) remained, however the fish were at a similar developmental stage.

Following field collections, fish were transported to the Moss Landing Marine Laboratories (MLML) aquarium facility, where they were acclimated for a 2–3 week period in flow-through seawater at ambient temperature (10–12°C), pH ~8.0, and 12:12 photoperiod. Fish were fed frozen mysids daily *ad libitum*. Fish were then individually tagged using visible implant elastomer tags (Northwest Marine Technologies, Inc.), using a unique color combination and placement of the dye near various body landmarks (e.g., head, tail, dorsal musculature, left vs. right side, etc.). Lengths were measured at the beginning and end of the experiments to the nearest 0.1 mm and weights were measured prior to respirometry studies and at the end of the study period. Juveniles were immature and could not be sexed.

### Rearing conditions and *p*CO_2_ manipulation

Following acclimation, fish were transported to the Monterey Bay Aquarium Research Institute (MBARI) and placed in four *p*CO_2_ treatments, simulating the predicted effects of ocean acidification (*n* = 34 copper rockfish and *n* = 34 blue rockfish) in a flow through seawater system. Seawater pH was reduced by bubbling CO_2_ gas into seawater through a Liqui-Cel membrane contactor to maintain *p*CO_2_ treatments in four large header tanks (500 L) corresponding to four pH levels: ambient pH ~7.9 (*p*CO_2_ ~500 μatm), pH = 7.7 (*p*CO_2_ ~750 μatm), pH = 7.5 (*p*CO_2_ ~1900 μatm), and pH = 7.3 (*p*CO_2_ ~2800 μatm), given predictions for the next 100 years and beyond. The most extreme pH treatments reflect conditions that already occur for short durations at our Carmel Bay collection site during intense upwelling (S1 Fig). Due to logistical and space restrictions in the lab, treatment water was then delivered to a single 100 L experimental tank per treatment containing *n* = 8–10 juvenile rockfish of a particular study species, at a flow rate of 0.5 L min^-1^. Subsequent experiments on other juvenile rockfish with multiple tanks per treatment demonstrated no compelling effect of tank or tank position on the response variables measured (H.W. Fennie, *unpublished data*). Temperature was maintained at constant 10°C using a heater and chiller connected with a counter current exchanger. Dissolved oxygen (DO) was maintained near 100% saturation (~8–9 mg L^-1^) using a similar membrane contactor and O_2_ gas. Flow of CO_2_ and O_2_ into the header tanks was controlled by solenoid valves and mass flow controllers (Sierra Instruments, SmartTrak 100) based on feedback from changes in pH levels measured using a Honeywell Durafet pH sensor and dissolved oxygen levels using Aanderaa AADI oxygen optodes (model 3830) connected to a computer monitoring and control system running LabView software. A porthole over each rearing tank was used to deliver food (frozen mysids) and provide access for daily measurements of temperature, pH and DO in each tank using a Hach HQ40d portable multi-parameter meter. Every two weeks, water samples were collected from one tank in each treatment and analyzed for dissolved inorganic carbon (DIC) on a custom-built DIC analyzer [44], total alkalinity (A_T_) using an alkalinity titrator (SI Analytics, TitroLine 7000), and pH using an pH spectrophotometer (Shimadzu, UV-1601), following standard protocols [45]. These metrics were used to verify our pH measurements and calculate CO_2_ concentration (*p*CO_2_) using the program CO2SYS [46].

### Critical swimming speed

Swimming physiology tests commenced after 5–8 weeks of treatment exposure for copper rockfish and 7–9 weeks for blue rockfish (Table 2). For each species, individuals were selected randomly and tested for critical swimming speed in a swimming flume (Loligo Systems model 10), consisting of a 10 L respirometer inside a 400 L buffer tank to maintain water temperatures. Flow rates are adjusted by increasing power to a motorized impeller and water velocities were validated using fluorescent tracer dye. After excluding individuals that refused to swim in the flume, the final samples sizes were *n* = 25 for copper rockfish and *n* = 30 for blue rockfish.

Critical swimming speed (U_crit_) is a measure of maximum performance during a short-term sustained swimming challenge. Scaled to body length, U_crit_ can be used to compare relative swimming performance and maximum aerobic capacity among fishes of differing lengths and body forms [29]. For each trial, an individual fish was randomly selected and transferred to the swim tunnel and allowed to acclimate for 15 min (Table 2). Following methods used previously to test swimming capabilities of juvenile rockfish [30], we then increased the flow rate by approximately one body length (BL) per second every 2 min until the subject fatigued and could no longer maintain position in the flume for the full 2 min. U_crit_ was then calculated as: U_crit_ = U_i_ + U(t/t_i_), where, U_i_ is highest velocity maintained for the whole interval (penultimate speed), U = velocity increment (1 BL s^-1^), t = time elapsed at fatigue velocity, and t_i_ = set time interval for each velocity increment (2 min). These methods have been used successfully to document the ontogenetic development of swimming performance from birth through juvenile stages of multiple rockfish species [30].

### Behavioral lateralization

After approximately 10 weeks of cumulative treatment exposure, we tested changes in brain functional asymmetry and behavioral lateralization using a detour test with a double T-Maze. The degree of individual lateralization (bias for left vs. right turning decisions) can affect performance in cognitive tasks, schooling behavior, spatial orientation, and escape reactions from predators, and lateralization has been shown to change in response to elevated *p*CO_2_ [11,18]. Thus, behavioral lateralization provides a powerful test of brain function for different decision-making tasks involving left versus right responses to environmental stimuli. After a minimum of 1 week recovery from the U_crit_ experiment and after a total of 10 weeks of treatment exposure (Table 2), randomly selected fish were introduced into a two-way T-maze (modified 50×30×25 cm L×W×H aquarium) and acclimated for 5 min. Following methods in ref. [11], we used a small hand net to coax each fish to swim down a channel (30×10 cm L×W) until reaching a dead end, at which point they had to turn left or right. The hand net was used at the beginning of each individual’s swim down the channel and then was stopped at the halfway point and left in place to avoid interference with the subsequent turning decision. Ten consecutive tests were conducted for each fish. To account for possible asymmetry in the setup that may affect turn bias, tests were carried out alternately starting from the two ends of the channel. In order to compare differences in lateralization among treatment groups, we calculated a relative lateralization index (*L*_R_): [(# left turns–# right turns) / (# left turns + # right turns)]*100. Mean *L*_R_ was used to assess turning bias at the population level. We also calculated the absolute lateralization index (LA): [absolute value (# left turns–# right turns) / (# left turns + # right turns)]*100 to test for turn bias regardless of the turn direction. After excluding individuals that refused to swim the full length of the flume after coaxing, sample sizes were *n* = 29 for copper rockfish and *n* = 33 for blue rockfish.

### Respirometry and aerobic scope

After fish had a minimum 3 weeks recovery from behavioral lateralization tests, and following approximately 15 weeks of cumulative treatment exposure (Table 2), we recorded routine (or standard) metabolic rates and maximal O_2_ uptake to estimate aerobic capacity [12] by measuring oxygen consumption rates before and during swimming following the techniques reported in ref. [47]. Fish were blotted and wet weighed to the nearest 0.1 mg to obtain weights for mass-specific respiration calculations and then were fasted for 24 hrs. After fasting, fish were transferred to the Loligo 10L swim flume for 2 hrs to acclimate prior to starting a test. In preliminary trials we found O_2_ consumption did not change with longer acclimation periods (up to 24 hrs). For routine metabolic rates, we recorded oxygen consumption by measuring dissolved oxygen values continuously using an oxygen optode (FireSting O_2_ sensor, PyroScience) for 1 hr. In these tests, the flow rate was kept to a minimal level (lowest setting of the mechanical motor; 0 hz, <0.4 cm/s) to encourage mixing of the water in the swim chamber, but at a level that did not influence the ability of fish to rest motionless at the bottom of the chamber. Next, we determined maximum oxygen consumption by setting the flow rate one step below (i.e., 1 BL s^-1^ slower than) the maximum sustained swimming speed determined in the previous U_crit_ trials for each individual. Changes in water O_2_ levels were then recorded for 20 min while the fish was swimming at its presumed maximal rate. After excluding individuals that refused to swim in the flume, the final sample sizes were *n* = 29 for copper rockfish and *n* = 34 for blue rockfish. Aerobic scope was calculated as the difference between maximum and resting O_2_ consumption. At the conclusion of the aerobic scope trials, the fish were immediately returned to their treatment tanks and allowed to recover for a minimum of 2 weeks (Table 2). They were then sacrificed after 21 weeks of total exposure in treatment conditions to obtain white muscle tissue for gene expression analysis.

### Statistical analysis

To test for significant differences in behavioral and physiological response variables as a function of *p*CO_2_ treatment, we used single factor Analysis of Variance (ANOVA). To identify which *p*CO_2_ treatment levels were significantly different from each other, we conducted multiple comparison tests using the Tukey HSD post-hoc test. Data were tested to ascertain whether they violated any of the statistical model assumptions (i.e., independence, normality, etc.), prior to conducting the ANOVAs. We used a principal components analysis to examine correlations in the responses of performance measures across the individuals subjected to each of the experimental trials throughout the time course of the experiment. Subjects were grouped into the two lowest and two highest *p*CO_2_ treatments to examine evidence for clustering of response measures as a function of *p*CO_2_ exposure history.

### Dissections and tissue storage

Fish were sacrificed to obtain white muscle tissue for biochemical and gene expression analyses following 2 weeks of a resting period in the appropriate treatment conditions following the aerobic scope trials. To eliminate potential metabolic changes due to specific dynamic action, fish were starved for 24 hrs prior to being euthanized. For each individual, muscle, brain, gill, liver and otoliths were dissected and tissues were flash frozen in liquid nitrogen within 5–10 min. Frozen tissues were stored at -80°C for subsequent analyses. A subsample of fish (run previously through each performance trial), were selected randomly for biochemical and gene expression analyses. We analyzed differential gene expression in *n* = 15 copper rockfish (*n* = 4 fish per treatment except the extreme CO_2_, which had *n* = 3) and *n* = 10 blue rockfish (*n* = 3 fish per treatment except the moderate and extreme CO_2_, which had *n* = 2). We measured metabolic enzyme activity in *n* = 8 fish in the control and extreme *p*CO_2_ treatments for both species (intermediate treatments were not tested).

### Enzyme activity assays

Muscle tissue samples were diluted 10× in homogenization buffer (50 mM KPO4, pH 6.8 at 20°C) and homogenized with a Qiagen Tissue Lyser using a 5 mm stainless steel bead for 4 min. at 50 Hz (Qiagen, Valencia, CA, USA). Homogenates were then centrifuged at 4°C for 10 min at 13,000 g and the resulting supernatants were serially diluted to 100× (CS) or 200× (LDH). Analyses were performed in a temperature controlled TECAN Infinite^®^ M200 microplate reader at 30°C ± 0.2. Tissue homogenates for the same individual were assayed for CS in triplicate and LDH in quadruplicate on the same day. Average activity was calculated in international units per gram fresh weight.

Citrate synthase (CS) (EC. 4.1.3.7) activity. Production of coenzyme A-SH in crude tissue homogenates was monitored via an increase in absorbance of dithionitrobenzoic acid (DTNB) at 412 nm. The reaction mixture consisted of 25.5 ml of assay buffer (50 mM imidazole/HCl, at pH 8.2 at 20°C), 3.0 ml of 15 mM MgCl_2_, 1.5 mg of DTNB and 3.0 mg of acetyl-CoA. The CS reaction was initiated by vigorously mixing 5 μl of homogenate to 195 μl of reaction mixture with oxaloacetate (OAA) from a stock solution of 53 mg OAA in 10 ml of assay buffer. Controls were run in triplicate by adding 5 μl of homogenate to 195 μl of reaction mixture without OAA. Absorbance was measured for 20 min using the kinetic cycle setting with a shaking duration of 5s in orbital mode (amplitude 2.5 mm) and 3 flashes per read. The linear rate of change in absorbance of the control wells was subtracted from that of the experimental wells before calculating average activity.

Lactate dehydrogenase (LDH) (EC 1.1.1.27) activity. The conversion of NADH to NAD^+^ was monitored via a decrease in absorbance at 340 nm. NADH (0.15 mM) and sodium pyruvate (0.2 mM) were vigorously mixed with assay buffer (0.20 M imidazole/HCl at pH 7.3 at 20°C) into a total volume of 200 μl. Absorbance was measured for 10 min using the kinetic cycle setting with a shaking duration of 5 s in orbital mode (amplitude 2.5 mm) and 3 flashes per read.

### cDNA library preparation

Total RNA was subsequently extracted from frozen white dorsal muscle tissue samples (*n* = 4 per treatment) using a Qiagen RNeasy kit (Qiagen, Valencia, CA; cat. no. 74104) following standard protocol. RNA quality was examined with a Nanodrop spectrophometer and 1% agarose gel; RNA was quantified using a Qubit 2.0 fluorometer using the RNA BR assay kit (cat. no. Q10210). High quality RNA was defined by Nanodrop 260/280 ratios between 2.0–2.2, and no degradation on the gel. mRNA was isolated from 1 μg of total RNA from each sample using a poly(A) mRNA magnetic isolation kit (New England Biolabs, Inc.; cat. no. E7490S).

cDNA libraries were constructed using the NEBNext Ultra Directional RNA Library kit for Illumina (NEB; cat no. E7420). NEBNext adaptors and index primers were ligated to libraries for multiplex up to 12 samples on a single lane (NEB; cat no. E7335S). Individual libraries were examined for size distribution and concentration using a Bioanalyzer at the U.C. Berkeley QB3 Vincent Coates Genomics Sequencing Laboratory. The sequencing facility used Qubit and qPCR to determine final cDNA library concentration. High quality libraries were loaded in equal amounts on to the flow cell and sequenced on an Illumina HiSeq2000 machine (Illumina, Inc.). For copper rockfish, seven individuals were randomly multiplexed into one 100 base pair (bp) paired end lane and eight individuals into one 100 bp single end lane. For blue rockfish, 12 individuals were multiplexed into one 100 bp paired end lane. All rockfish libraries were prepared and sequenced for each species at the same time.

### Transcriptome assembly and annotation

NEB adaptor sequence and bases with a PHRED quality score <20 were removed from the ends using the FASTX toolkit (http://hannonlab.cshl.edu/fastx_toolkit/). Reads <20 bp were discarded. All copper rockfish (*n* = 15) QC reads were assembled into a *de novo* transcriptome assembly using Trinity following an *in silico* normalization step within Trinity [48]. Heatmaps were generated for DE genes using the package gplots in R with hierarchical clustering (Euclidean distance) to group genes by similar expression profiles (Fig 3A). Open reading frames (ORFs) were translated into peptide sequences (TransDecoder; http://transdecoder.sourceforge.net/) and annotated against an NCBI non-redundant database for teleost fish and SwissProt using blastp.

### Mapping and differential gene expression analysis

Individual blue and copper rockfish sequences were aligned and mapped to the copper rockfish *de novo* assembly using RSEM v1.2.11 (RNA-Seq by Expectation Maximization; ref. [49]) using the bowtie alignment method implemented in Trinity (r2013-11-10) (S1 Table). We conducted an unsupervised PCA analysis of all contigs in R to show variation within and among treatments before performing differential gene expression analysis. The empirical analysis of digital gene expression data (edgeR) Bioconductor package was used to identify differentially expressed genes using the p-value cutoff for false discovery rate (FDR) of <0.001 and a minimum of a 1.5-fold expression cut-off among pH treatments [50]. edgeR is recommended for small sample sizes [50], but can be slightly liberal with default FDR settings [51], thus we erred on the side of a more conservative FDR cut-off. edgeR includes a TMM (trimmed mean of M-values) scaling normalization that helps account for differences in total cellular RNA production across all samples [48]. Dispersion values were calculated using the replicate groups and exact tests utilizing a negative binomial distribution were used to identify differentially expressed transcripts and genes among all pairwise groups [50]. To group significant genes by gene ontology categories, we input UniProt accession numbers (S4 Table) into the Protein Analysis Through Evolutionary Relationships (PANTHER v9.0) Classification System ([52]; http://www.pantherdb.org/).

### Data accessibility

Genomics data are available through the NCBI database (http://www.ncbi.nlm.nih.gov/), including the copper rockfish *de novo* transcriptome (BioSample: SAMN03757544) and individual gene expression sample files (SRA: SRS951361).

## Supporting Information

S1 Table Summary of copper rockfish (*Sebastes caurinus*) Trinity *de novo* assembly and BLAST annotations. (PDF)

S2 Table Copper rockfish differentially expressed (DE) genes grouped by heatmap cluster (Fig 3A), including manual annotation (category) based on gene ontology classification and primary literature review, Uniprot gene description, accession and e-value, maximum fold change, Trinity contig, and whether the Uniprot Accession was significant in both species. This list includes 93 annotated genes (of the 147 total DE genes). Pairwise significance is indicated by: 1 = 3200 *vs*. 500 μatm; 2 = 3200 *vs*. 800 μatm; 3 = 2000 *vs*. 500 μatm; 4 = 3200 *vs*. 2000 μatm; 5 = 2000 *vs*. 800 μatm; 6 = 800 *vs*. 500 μatm.
(PDF)

S3 Table Blue rockfish differentially expressed (DE) genes grouped by heatmap cluster (Fig 3A), including manual annotation (category) based on gene ontology classification and primary literature review, Uniprot gene description, accession and e-value, maximum fold change, Trinity contig, and whether the Uniprot Accession was significant in both species. This list includes 242 annotated genes (of the 358 total DE genes). Pairwise significance is indicated by: 1 = 3200 *vs*. 500 μatm; 2 = 3200 *vs*. 800 μatm; 3 = 2000 *vs*. 500 μatm; 4 = 3200 *vs*. 2000 μatm; 5 = 2000 *vs*. 800 μatm; 6 = 800 *vs*. 500 μatm.
(PDF)

S4 Table Gene Ontology (GO) consortium accession numbers from Fig 2B in the main text. (PDF)

S1 Fig pH time series (A) from Carmel Bay, California collected using a SeaFET sensor (with a Durafet ISFET pH electrode) deployed to the benthos in the center of a large kelp bed at 12 m depth (B). pH readings were made every 15 minutes and are plotted as hourly means. The gap from May to October 2013 occurred due to a flooded housing.
(PDF)

S2 Fig Absolute lateralization index (higher values are more lateralized) for copper and blue rockfish as a function of *p*CO_2_ treatment history. Bars are mean values (± SE). Letters over bars represent results of Tukey HSD post-hoc tests; significantly different means do not share letters in common.
(PDF)

S3 Fig Growth rates (mm d^-1^) measured as a change in length over the experimental exposure duration for copper and blue rockfish as a function of *p*CO_2_ treatment history. Bars are mean values (± SE). Letters over bars represent results of Tukey HSD post-hoc tests; significantly different means do not share letters in common.
(PDF)

S4 Fig Routine metabolic rate and maximum metabolic rate of copper and blue rockfish. (A,B) Routine metabolic rate was measured as the oxygen consumption rate while at rest. (C,D) Maximum metabolic rate was measured as the oxygen consumption rate calculated while the fish swam at its presumed maximum rate in a swim tunnel. Letters over bars represent results of Tukey HSD post-hoc tests; significantly different means do not share letters in common.
(PDF)

S5 Fig Principal component analysis using an unsupervised analysis of all gene transcripts. Treatment groupings are similar to those observed in the heatmaps for differentially expressed gene patterns (Fig 3). A) For copper rockfish, PC2 (12.9%) and PC3 (11.6%) separated *p*CO_2_ treatments into the two highest and lowest *p*CO_2_ treatments. The variance explained by PC1 (13.8%) did not differ greatly from PC2 and PC3. B) For blue rockfish, PC1 (18.3%) and PC2 (13.8%) separated the control and extreme *p*CO_2_ treatments and clustered the moderate treatments.
(PDF)

S6 Fig Molecular function and biological process Gene Ontology (GO) categories represented in the annotated copper rockfish *de novo* transcriptome assembly. Compared to the full assembly, differentially expressed genes (Fig 3B) showed increased expression of genes involved in transcription activity and biological regulation in copper rockfish and increased expression of genes involved in structural molecule activity in blue rockfish.
(PDF)
