# Supplementary material for: Species-Specific Responses of Juvenile Rockfish to Elevated pCO2: From Behavior to Genomics
Source: PLoS One. 2017 Jan 5;12(1):e0169670. doi: 10.1371/journal.pone.0169670 (PMC5215853; doi:10.1371/journal.pone.0169670)
Supplement: S1 Table — (PDF) [file pone.0169670.s001.pdf]

S1 Table. Summary of copper rockfish (*Sebastes caurinus*) Trinity *de novo* assembly and BLAST annotations.

Total contigs ("genes")	56,992
Total isoforms	90,804
Total bp in assembly	121,945,631
N50	2,536
Minimum contig length	201
Maximum contig length	59,752
Median contig length	711
Average contig length	1,343
SD contig length	1,571
Total contigs with match to SwissProt	30,059 (52.7%)
