# Supplementary material for: Species-Specific Responses of Juvenile Rockfish to Elevated pCO2: From Behavior to Genomics
Source: PLoS One. 2017 Jan 5;12(1):e0169670. doi: 10.1371/journal.pone.0169670 (PMC5215853; doi:10.1371/journal.pone.0169670)
Supplement: S2 Table — This list includes 93 annotated genes (of the 147 total DE genes). Pairwise significance is indicated by: 1 = 3200 vs. 500 μatm; 2 = 3200 vs. 800 μatm; 3 = 2000 vs. 500 μatm; 4 = 3200 vs. 2000 μatm; 5 = 2000 vs. 800 μatm; 6 = 800 vs. 500 μatm. (PDF) [file pone.0169670.s002.pdf]

S2 Table. Copper rockfish differentially expressed (DE) genes grouped by heatmap cluster (Fig 3A), including manual annotation (category) based on gene ontology classification and primary literature review, Uniprot gene description, accession and e-value, maximum fold change, Trinity contig, and whether the Uniprot Accession was significant in both species. This list includes 93 annotated genes (of the 147 total DE genes). Pairwise significance is indicated by: 1 = 3200 *vs.* 500 µatm; 2 = 3200 *vs.* 800 µatm; 3 = 2000 *vs.* 500 µatm; 4 = 3200 *vs.* 2000 µatm; 5 = 2000 *vs.* 800 µatm; 6 = 800 *vs* .500 µatm.

Heatmap cluster	Category	UniProt Gene Name (Enzyme Code)	Max Fold Change	Trinity Contig	Uniprot Acc.	E-value	Both spp. ?	Significant Pairwise Comparison			
a	Cell differentiation	Transmembrane protein 100	2.2	comp47156	Q569C0	0.00005	X	5			
a	Electron transport chain	Cytochrome c oxidase subunit 7A2, mitochondrial	2.5	comp37703	P48771	3E-30		3			
a	Electron transport chain	NADH dehydrogenase [ubiquinone] 1 alpha subcomplex subunit 4 (EC:1.9.3.1; EC:1.6.5.3)	4.3	comp14763	Q6PBH5	6E-43	X	1	2	3	5
a	Metabolism, carbohydrate - gluconeogenesis	[Pyruvate dehydrogenase (acetyl-transferring)] kinase isozyme 2, mitochondrial	2.7	comp54678	Q15119	6E-121	X	3	5		
a	Metabolism, carbohydrate - gluconeogenesis	L-serine dehydratase/L-threonine deaminase (EC:4.2.1.17)	4.8	comp51936	Q8VBT2	2E-115		3			
a	Muscle contraction	Myosin heavy chain, fast skeletal muscle	3.2	comp57572	Q90339	3E-20	X	3	5		
a	Muscle contraction	Myosin heavy chain, fast skeletal muscle	2.7	comp51509	Q90339	2E-29	X	3			
a	Muscle contraction	Troponin T, cardiac muscle isoforms	3.1	comp41695	P02642	1E-65		3			
a	NA	Protein slowmo homolog 2	4.3	comp53799	Q4R5S9	1E-86	X	3	5		
a	NA	Uncharacterized protein C11orf96 homolog	2.7	comp47627	A8IHN8	4E-12		3	5		
a	Oxidation reduction	L-threonine 3-dehydrogenase, mitochondrial (EC:1.1.1.103)	2.7	comp53091	Q2KIR8	1E-17		3	5		
a	Proteolysis	OTU domain-containing protein 1	5.2	comp42241	Q9CUB6	8E-102		1	2	3	5
a	Stress response - oxidative stress	Mitochondrial uncoupling protein 2 (UCP2)	2.5	comp52795	P70406	1E-82	X	3	5		
a	Transcription	Hypermethylated in cancer 2 protein	6.5	comp51956	Q90W33	5E-16	X	3			
b	Actin cytoskeleton organization	Xin actin-binding repeat-containing protein 1	2.4	comp52428	Q5PZ43	0	X	3			
b	Carbohydrate transport	Solute carrier family 2, facilitated glucose transporter member 4	2.6	comp53718	Q27994	0	X	3			
b	Electron transport chain	Cytochrome b-c1 complex subunit 10	2.5	comp44435	Q9CPX8	9E-19		1			
b	Electron transport chain	NADH dehydrogenase [ubiquinone] iron-sulfur protein 2, mitochondrial (EC:1.6.5.3, EC:1.6.99.3)	2.2	comp47814	P17694	0		1			
b	Electron transport chain	NADH dehydrogenase [ubiquinone] iron-sulfur protein 7, mitochondrial (EC:1.6.5.3, EC:1.6.99.3)	2.2	comp41545	P0CB84	4E-103		1	3		
b	Heme biosynthesis	ATPase inhibitor B, mitochondrial	2.7	comp47335	Q1LYB6	2E-33		3			
b	Ion transport - chloride	Glycine receptor subunit alpha-4	2.7	comp49601	Q61603	2E-132		3	5		
b	Metabolism, lipid	Prostaglandin E synthase	2.0	comp48620	Q8HZJ2	6E-59		5			
b	NA	Inositol 1,4,5-trisphosphate receptor-interacting protein	1.6	comp50156	Q90XY5	0		2	5		
b	Oxidation reduction	Type III iodothyronine deiodinase	2.3	comp44295	A7YD35	9E-166		5			
b	Signaling	Ephrin-A1	1.7	comp49567	P52794	1E-64		5			
b	Signaling	Ral guanine nucleotide dissociation stimulator-like 1	1.5	comp53817	Q6P112	0		2	5		
b	Transcription	Krueppel-like factor 2	1.5	comp41845	Q60843	1E-65	X	5			
b	Transcription	Krueppel-like factor 2	1.5	comp37588	Q9Y5W3	1E-65		5			
b	Transcription	TSC22 domain family protein 3	2.5	comp46933	Q5RED5	3E-50		3	5		
b	Transport (sodium-phosphate symporter)	Sodium-dependent phosphate transporter 1-B	1.9	comp49122	Q6PFM1	6E-34		5			
b	Transport (sodium-phosphate symporter)	Sodium-dependent phosphate transporter 1-B	1.8	comp48192	Q6PFM1	0		5			

Heatmap cluster	Category	UniProt Gene Name (Enzyme Code)	Max Fold Change	Trinity Contig	Uniprot Acc.	E-value	Both spp. ?	Significant Pairwise Comparison			
**c**	Angiogenesis	Plasminogen activator inhibitor 1	1.8	comp52034	P13909	0.0000002		5			
**c**	Angiogenesis, Apoptosis	Angiopoietin-related protein 4	1.6	comp52633	Q2KJ51	2E-95		5			
**c**	Apoptosis	Apoptosis facilitator Bcl-2-like protein 14 (BCL2L14)	2.4	comp51993	Q9BZR8	2E-10		5			
**c**	Apoptosis	NF-kappa-B inhibitor alpha (NFKBIA)	2.7	comp46291	P25963	6E-75		2	5		
**c**	Carbohydrate binding	L-rhamnose-binding lectin CSL2	5.0	comp45362	P86178	4E-57		6			
**c**	Immune	C-type lectin domain family 4 member M	1.6	comp54364	Q8HY10	7E-17		3			
**c**	Immune	Pleurocidin	1.8	comp48163	Q90ZY0	0.000001		3			
**c**	Immune	Proteinase-activated receptor 2	2.9	comp53848	P55086	1E-122		2	5		
**c**	Immune	Pyrin	1.9	comp44188	O15553	7E-10		3			
**c**	Metabolism, lipid - steroid biosynthesis	3 beta-hydroxysteroid dehydrogenase type 7 (EC:1.1.1.181)	3.3	comp56723	O35048	3E-13		1			
**c**	mRNA metabolism	Zinc finger protein 36, C3H1 type-like 2-A	2.4	comp46780	Q7ZXW9	9E-28		5			
**c**	NA	GSK-3-binding protein	1.9	comp53985	O93343	9E-23		6			
**c**	NA	Immunoglobulin-like and fibronectin type III domain-containing protein 1	2.6	comp56663	Q86VF2	0	X	2			
**c**	NA	Kelch-like protein 38	2.3	comp53727	Q1LYM6	3E-14		5			
**c**	NA	Otopetrin-2	1.7	comp40619	Q80SX5	7E-148		6			
**c**	NA	Tripartite motif-containing protein 47	2.7	comp52368	Q96LD4	1E-28		5			
**c**	Proteolysis	A disintegrin and metalloproteinase with thrombospondin motifs 1 (EC:3.4.24)	2.5	comp56293	Q9WUQ1	1E-171		5			
**c**	Signaling	ADP-ribosylation factor-like protein 5B	2.0	comp51118	Q9D4P0	5E-84		2	5		
**c**	Signaling	ERBB receptor feedback inhibitor 1	2.0	comp53814	Q99JZ7	2E-79		2	5		
**c**	Signaling	Macrophage mannose receptor 1	2.3	comp53136	P22897	7E-15		3			
**c**	Signaling	Protein-glutamine gamma-glutamyltransferase 5 (EC:2.3.2.13)	3.9	comp51999	O43548	2E-148	X	6			
**c**	Signaling	Proteinase-activated receptor 1	1.9	comp51339	P47749	2E-74		3	5		
**c**	Signaling	Regulator of G-protein signaling 13	2.3	comp48692	O14921	7E-37		2	5		
**c**	Signaling	Regulator of G-protein signaling 2	3.4	comp50723	P41220	6E-49		2	5		
**c**	Signaling	Synaptosomal-associated protein 25-A	14.3	comp47266	Q5TZ66	2E-136		5			
**c**	Signaling, Apoptosis	Protein NLRC3	2.0	comp49488	Q5DU56	0.000000005		6			
**c**	Signaling, Apoptosis, Ubiquitination	Suppressor of cytokine signaling 3	3.7	comp47632	Q90X67	2E-78		3	5		
**c**	Signaling, Cell growth	Insulin-like growth factor-binding protein 1	2.3	comp37415	P24591	4E-39		2	5		
**c**	Stress response - DNA damage, apoptosis	Growth arrest and DNA damage-inducible protein GADD45 beta	2.5	comp47491	O75293	0.00001		5			
**c**	Stress response - molecular chaperone	Heat shock 70 kDa protein (Hsp70)	5.2	comp48901	Q91233	4E-97	X	5	6		
**c**	Stress response - oxidative stress	NADPH oxidase organizer 1 (NOXO1)	3.2	comp56909	Q8NFA2	1E-55		6			
**c**	Stress response, Cell Cycle	Dual specificity protein phosphatase 1 (Dusp1)	2.4	comp50371	P28562	9E-88		2	3	5	
**c**	Stress response, Cell Cycle	Dual specificity protein phosphatase 2 (Dusp2)	2.7	comp46968	Q05922	2E-92		2			
**c**	Structural	Plasticin	15.3	comp49700	P31393	0		1	4		
**c**	Transcription	AT-rich interactive domain-containing protein 3A	4.0	comp52519	A2BEA6	2E-38		2	3	5	
**c**	Transcription	cAMP-responsive element modulator	1.9	comp14888	P79145	6E-25		5			
**c**	Transcription	Chromobox protein homolog 7	1.8	comp51571	O95931	1E-39		3	5		
**c**	Transcription	Class E basic helix-loop-helix protein 40	1.6	comp52127	O35185	2E-126		5	6		
**c**	Transcription	CLOCK-interacting pacemaker	1.5	comp55156	Q9C0C6	5E-12		6			

Heatmap cluster	Category	UniProt Gene Name (Enzyme Code)	Max Fold Change	Trinity Contig	Uniprot Acc.	E-value	Both spp. ?	Significant Pairwise Comparison			
**c**	Transcription	Cysteine/serine-rich nuclear protein 1	2.4	comp55607	Q96S65	2E-84		2	5		
**c**	Transcription	Early growth response protein 3	1.8	comp50912	Q06889	6E-19		5			
**c**	Transcription	Fos-related antigen 1	5.5	comp47646	P10158	2E-45		5			
**c**	Transcription	Heterogeneous nuclear ribonucleoprotein D0	2.1	comp47849	Q60668	8E-103		3			
**c**	Transcription	Jun dimerization protein 2	3.3	comp48364	Q78E65	3E-31		2	5		
**c**	Transcription	Krueppel-like factor 4	3.3	comp45001	Q60793	5E-69		2	5		
**c**	Transcription	Nuclear factor interleukin-3-regulated protein	2.0	comp47695	Q66J36	5E-18		5			
**c**	Transcription	Nuclear factor interleukin-3-regulated protein	1.6	comp52097	Q5FW38	1E-29		5			
**c**	Transcription	Nuclear receptor subfamily 1 group D member 2	1.9	comp54949	Q14995	5E-90	X	5	6		
**c**	Transcription	Nuclear receptor subfamily 4 group A member 1	2.0	comp54573	Q04913	0		5			
**c**	Transcription	Proto-oncogene c-Fos	4.3	comp44764	P53450	2E-128		2	5		
**c**	Transcription	Src-like-adapter 2	1.8	comp49071	Q8R4L0	8E-62		2	5		
**c**	Transcription	Transcription factor jun-B	3.0	comp42088	P79703	7E-116		5			
**c**	Transcription	Transcription factor jun-B	4.2	comp37271	P79703	2E-77		2	5		
**c**	Transcription	Transcription factor jun-D	2.8	comp37506	P27921	2E-69		2	3	5	
**c**	Transcription	Transcription factor MafK	1.7	comp53147	Q90596	2E-57		3	5		
**c**	Transcription	Transcription factor VBP	1.7	comp51898	Q92172	1E-43		3			
**c**	Transcription	Transcriptional regulator Myc	3.0	comp48764	P49709	6E-42		5			
**c**	Transcription, Stress response	CCAAT/enhancer-binding protein beta (C/EBPB)	3.4	comp14945	P17676	2E-30		5			
**c**	Transcription, Stress response	CCAAT/enhancer-binding protein delta (C/EBPD)	3.2	comp41824	P49716	3E-53	X	2	5	6	
**c**	Transport	Calcium-binding mitochondrial carrier protein SCaMC-2-A	1.9	comp53573	Q6NYZ6	1E-51		5			
**c**	Ubiquitination	E3 ubiquitin-protein ligase NEURL3 (UBE2E1)	4.3	comp50303	Q96EH8	4E-27		2	5		
**c**	Ubiquitination	Ubiquitin-conjugating enzyme E2 H (UBE2H)	1.6	comp55565	P62257	9E-91		3	5		
