# Supplementary material for: Species-Specific Responses of Juvenile Rockfish to Elevated pCO2: From Behavior to Genomics
Source: PLoS One. 2017 Jan 5;12(1):e0169670. doi: 10.1371/journal.pone.0169670 (PMC5215853; doi:10.1371/journal.pone.0169670)
Supplement: S3 Table — This list includes 242 annotated genes (of the 358 total DE genes). Pairwise significance is indicated by: 1 = 3200 vs. 500 μatm; 2 = 3200 vs. 800 μatm; 3 = 2000 vs. 500 μatm; 4 = 3200 vs. 2000 μatm; 5 = 2000 vs. 800 μatm; 6 = 800 vs. 500 μatm. (PDF) [file pone.0169670.s003.pdf]

S3 Table. Blue rockfish differentially expressed (DE) genes grouped by heatmap cluster (Fig 3A), including manual annotation (category) based on gene ontology classification and primary literature review, Uniprot gene description, accession and e-value, maximum fold change, Trinity contig, and whether the Uniprot Accession was significant in both species. This list includes 242 annotated genes (of the 358 total DE genes). Pairwise significance is indicated by: 1 = 3200 *vs.* 500 µatm; 2 = 3200 *vs.* 800 µatm; 3 = 2000 *vs.* 500 µatm; 4 = 3200 *vs.* 2000 µatm; 5 = 2000 *vs.* 800 µatm; 6 = 800 *vs.* 500 µatm.

Heatmap Cluster	Category	UniProt Gene Name (Enzyme Code)	Max Fold Change	Trinity Contig	Uniprot Acc.	E-value	Both spp. ?	Significant Pairwise Comparison			
**d**	Actin cytoskeleton organization, structural	Xin actin-binding repeat-containing protein 1	1.7	comp52428	Q5PZ43	0	X	3			
**d**	Actin cytoskeleton organization, structural	Xin actin-binding repeat-containing protein 2	1.9	comp56669	A4UGR9	0		3			
**d**	Aerobic respiration - TCA cycle	Isocitrate dehydrogenase [NAD] subunit gamma 1, mitochondrial (EC:1.1.1.41)	1.8	comp52991	P70404	0		1			
**d**	Apoptosis	Bcl-2/adenovirus E1B 19 kDa-interacting protein 2-like protein	2.5	comp54094	Q99JU7	4.00E-73		2	6		
**d**	Calcium ion binding	Calcineurin B homologous protein 3	4.6	comp45668	Q5U554	2.00E-76		6			
**d**	Calcium ion binding	Calcium-binding and coiled-coil domain-containing protein 1	2.2	comp53424	A2BGD5	3.00E-99		6			
**d**	Calcium ion binding	Calumenin-A	4	comp45928	B5X186	1.00E-91		2	6		
**d**	Calcium ion binding	Endosialin	1.9	comp54762	Q9HCU0	6.00E-65		3			
**d**	Calcium ion transport	Junctophilin-3	1.9	comp47707	Q8WXH2	4.00E-08		6			
**d**	Calcium ion transport, regulation of	Sarcolipin	2.8	comp51430	Q6SLE7	4.00E-06		1			
**d**	Carbohydrate transport	Solute carrier family 2, facilitated glucose transporter member 4	2.2	comp53718	Q27994	0	X	1			
**d**	Carboxylic acid transport	Neutral amino acid transporter B(0)	2.3	comp55876	O19105	6.00E-28		3			
**d**	Cell death	Stonustoxin subunit beta	2.6	comp49167	Q91453	2.00E-21		1	2		
**d**	Cell death	Stonustoxin subunit beta	2.4	comp47777	Q91453	5.00E-17		1			
**d**	Cell differentiation	Transmembrane protein 100	2.2	comp47156	Q569C0	5.00E-05	X	1			
**d**	Cell-Cell adhesion	Claudin-like protein ZF-A89	5.7	comp47402	Q9YH91	3.00E-75		2			
**d**	Cell-Cell adhesion	Desmocollin-2	3.7	comp54009	P55292	4.00E-139		2	6		
**d**	Electron transport chain	NADH dehydrogenase [ubiquinone] 1 alpha subcomplex subunit 4 (EC:1.9.3.1; EC:1.6.5.3)	4.5	comp14763	Q6PBH5	6.00E-43	X	1	4		
**d**	Immune	Class I histocompatibility antigen, F10 alpha chain	2.3	comp57399	P15979	5.00E-11		1			
**d**	Immune	Complement C1q subcomponent subunit A	2.2	comp41841	P31720	3.00E-32		3			
**d**	Immune	Complement C1q subcomponent subunit B	1.9	comp51178	Q2KIV9	8.00E-18		3			
**d**	Immune	Complement C1q-like protein 4	4.8	comp41709	Q4ZJM9	7.00E-11		3			
**d**	Immune	Gamma-interferon-inducible lysosomal thiol reductase	1.8	comp15021	A6QPN6	2.00E-58		3			
**d**	Immune	Protein unc-93 homolog B1	1.5	comp46315	Q8VCW4	0		1			
**d**	Ion homeostasis - iron	Transferrin receptor protein 1	2.3	comp56625	Q5RDH6	0		3			
**d**	Ion transport	Bestrophin-3	2.9	comp55617	Q8N1M1	4.00E-121		1			
**d**	Ion transport - sodium	Sodium channel protein type 4 subunit alpha A	1.7	comp55883	Q2XVR7	2.00E-114		1			
**d**	Lipid transport	Cholesteryl ester transfer protein	2.1	comp48370	Q3V6R6	7.00E-128		3			
**d**	Metabolism, carbohydrate	Protein phosphatase 1 regulatory subunit 3C-B	4	comp48114	Q6P950	2.00E-39		2	6		
**d**	Metabolism, carbohydrate - gluconeogenesis	[Pyruvate dehydrogenase (acetyl-transferring)] kinase isozyme 2, mitochondrial (EC:2.7.11.2)	2.5	comp54927	Q15119	0	X	4			

Heatmap Cluster	Category	UniProt Gene Name (Enzyme Code)	Max Fold Change	Trinity Contig	Uniprot Acc.	E-value	Both spp. ?	Significant Pairwise Comparison			
**d**	Metabolism, carbohydrate - glycerol metabolism	Glycerol kinase (EC:2.7.1.30)	2.4	comp49633	Q63060	3.00E-178		1			
**d**	Metabolism, carbohydrate - glycolysis	6-phosphofructokinase, muscle type (EC:2.7.1.11)	2.1	comp57213	Q0IIG5	0		1			
**d**	Metabolism, carbohydrate - glycolysis	Glyceraldehyde-3-phosphate dehydrogenase (EC:1.2.1.12)	1.9	comp51658	Q5XJ10	0		1			
**d**	Metabolism, carbohydrate - glycolysis	Malate dehydrogenase, cytoplasmic (EC:1.1.1.37; EC:1.1.1)	1.5	comp15347	Q5ZME2	4.00E-29		3			
**d**	Metabolism, carbohydrate - TCA cycle	Aconitate hydratase, mitochondrial (EC:4.2.1.3)	3.3	comp55972	Q99KI0	0		1	1		
**d**	Metabolism, carbohydrate - TCA cycle	Aconitate hydratase, mitochondrial (EC:4.2.1.3)	3.3	comp37068	Q99798	6.00E-73		1			
**d**	Metabolism, lipid	Hydroperoxide isomerase ALOXE3	6.1	comp57380	Q9WV07	0		2	6		
**d**	Metabolism, lipid	Prosaposin	1.9	comp37362	P26779	3.00E-21		3			
**d**	Metabolism, lipid	Protein-cysteine N-palmitoyltransferase HHAT-like protein	2.9	comp55305	Q9HCP6	0		1	2	4	
**d**	Methylation	Methyltransferase-like protein 16 (EC:2.1.1)	2	comp53373	Q86W50	0		2			
**d**	Muscle contraction	Alpha-actinin-2	5.6	comp55547	P20111	5.00E-113		1			
**d**	Muscle contraction	Kelch-like protein 41 (EC:5.2.1.8; EC:1.1.1.95)	2.7	comp54920	O60662	0		1			
**d**	Muscle contraction	Myomesin-1	1.9	comp56282	P52179	3.00E-27		1			
**d**	Muscle contraction	Myosin heavy chain, fast skeletal muscle	6.3	comp57572	Q90339	3.00E-20	X	1			
**d**	Muscle contraction	Myosin heavy chain, fast skeletal muscle	5.5	comp50309	Q90339	6.00E-35	X	1			
**d**	Muscle contraction	Myosin heavy chain, fast skeletal muscle	8.5	comp51509	Q90339	2.00E-29	X	1	2		
**d**	Muscle contraction	Myosin heavy chain, fast skeletal muscle	6.3	comp42622	Q90339	5.00E-72	X	1	6		
**d**	Muscle contraction	Myosin heavy chain, skeletal muscle, adult	6.1	comp15143	P13538	2.00E-09		1			
**d**	Muscle contraction	Myosin heavy chain, skeletal muscle, adult	4.1	comp48507	P13538	2.00E-43		2			
**d**	Muscle contraction	Myosin light chain 3	5.7	comp37991	P16409	1.00E-84		1	3		
**d**	Muscle contraction	Myosin regulatory light chain 2, ventricular/cardiac muscle isoform	2.9	comp41556	P08733	2.00E-84		1			
**d**	Muscle contraction	Myosin-6	15.7	comp35082	Q02566	5.00E-34		1	3	6	
**d**	Muscle contraction	Myosin-7	5.1	comp45165	P11778	1.00E-66		1			
**d**	Muscle contraction	Myosin-7	6.5	comp30235	P49824	0		1	2	3	
**d**	Muscle contraction	Myosin-7	8.9	comp57464	Q8MJU9	2.00E-54		1	3		
**d**	Muscle contraction	Myosin-7	7.2	comp50662	P04461	7.00E-125		1	4	6	
**d**	Muscle contraction	Myosin-7	4.3	comp49763	P79293	4.00E-45		6	1		
**d**	Muscle contraction	Myosin-7B	6.8	comp52044	A2AQP0	0		1			
**d**	Muscle contraction	Myosin-binding protein C, cardiac-type	4	comp55329	Q90688	0		1	4		
**d**	Muscle contraction	Smoothelin	1.8	comp56015	P53814	2.00E-103		1			
**d**	Muscle contraction	Tropomyosin alpha-1 chain	5.3	comp56932	P13104	4.00E-39		1			
**d**	Muscle contraction	Tropomyosin alpha-1 chain	4.3	comp18996	P13105	2.00E-40		1			
**d**	Muscle contraction	Troponin I, fast skeletal muscle	5.4	comp36355	P02643	5.00E-34		1	3		
**d**	Muscle contraction	Troponin I, slow skeletal muscle	3.3	comp15250	P02645	3.00E-46		1	6		
**d**	Muscle contraction	Troponin T, fast skeletal muscle isoforms	2.7	comp44204	P12620	2.00E-13		1	3		
**d**	Muscle contraction, regulation of	Sarcoplasmic/endoplasmreticulum alcium ATPase 1 (EC:3.6.3.8)	7.1	comp30596	P70083	9.00E-117		1	2	6	
**d**	Muscle contraction, regulation of	Sarcoplasmic/endoplasmic reticulum calcium ATPase 1 (EC:3.6.3.8)	4.9	comp46584	P70083	0		1			
**d**	Muscle contraction, regulation of	Tropomyosin beta chain	4.6	comp37907	P58775	9.00E-48		1	6		
**d**	Muscle contraction, regulation of	Troponin C, slow skeletal and cardiac muscles	2.1	comp45843	P19123	4.00E-70		1			
**d**	Muscle contraction, regulation of	Troponin I, slow skeletal muscle	4.1	comp56738	Q9WUZ5	6.00E-35		1			

Heatmap Cluster	Category	UniProt Gene Name (Enzyme Code)	Max Fold Change	Trinity Contig	Uniprot Acc.	E-value	Both spp. ?	Significant Pairwise Comparison			
**d**	Muscle contraction, regulation of	Troponin I, slow skeletal muscle	3.3	comp42671	P19237	3.00E-62		1			
**d**	Muscle contraction, regulation of	Calsequestrin-2	3.9	comp42001	P19204	3.00E-175		1			
**d**	Muscle contraction,regulation of	Ryanodine receptor 3	1.7	comp47248	Q9TS33	3.00E-58		1			
**d**	Muscle tissue development	Cardiac phospholamban	1.9	comp55957	P26677	3.00E-08		1	4		
**d**	NA	Costars family protein	2.9	comp52866	Q6AVK1	1.00E-34		6			
**d**	NA	Immunoglobulin-like and fibronectin type III domain-containing protein 1	2.2	comp56663	Q86VF2	0	X	3			
**d**	NA	Protein slowmo homolog 2	2.6	comp53799	Q4R5S9	1.00E-86	X	1			
**d**	NA	UPF0692 protein C19orf54 homolog	2.4	comp50299	B0V3H4	7.00E-126		2			
**d**	Nucleotide metabolic process	Nicotinamide phosphoribosyltransferase (EC:2.4.2.19)	1.7	comp46498	Q80Z29	0		1			
**d**	Oxidation reduction	Putative ferric-chelate reductase 1	4.2	comp14794	A4QP81	2.00E-09		2			
**d**	Pprotein dehydration	Probable serine racemase	2.1	comp45394	Q54HH2	2.00E-32		4			
**d**	Proteolysis	A disintegrin and metalloproteinase with thrombospondin motifs 7	1.8	comp57118	Q9UKP4	0		3			
**d**	Proteolysis	Inactive serine protease 35 (EC:3.4.21)	1.8	comp37517	Q8C0F9	2.00E-78		3			
**d**	Proteolysis	Tryptase-2	2.2	comp15002	Q9XSM2	1.00E-49		3			
**d**	Proteolysis, endopeptidase	Calpain-9	3.2	comp53498	O14815	0		2	6		
**d**	RNA processing	U4/U6 small nuclear ribonucleoprotein Prp3	1.6	comp53338	Q5ZJ85	0		2			
**d**	Serine protease inhibitor	Kunitz-type protease inhibitor 2	4.1	comp44586	Q9WU03	6.00E-32		2	6		
**d**	Serine protease inhibitor	Pigment epithelium-derived factor	2.6	comp51278	Q95121	8.00E-81		1			
**d**	Signaling	Mitogen-activated protein kinase 14A (EC:2.7.11.24)	2.5	comp48171	Q90336	6.00E-160		6			
**d**	Signaling	NUAK family SNF1-like kinase 1 (EC:2.7.11)	1.9	comp47642	O60285	0		1			
**d**	Signaling	Poliovirus receptor-related protein 2	1.7	comp52619	P32507	2.00E-61		2			
**d**	Signaling	Protein Wnt-2b	2.6	comp53680	Q98SN7	0		1			
**d**	Signaling	Protein-glutamine gamma-glutamyltransferase 5 (EC:2.3.2.13)	4.4	comp51999	O43548	2.00E-148	X	1	2	5	6
**d**	Signaling	Ras GTPase-activating protein-binding protein 1	1.8	comp55108	Q32LC7	3.00E-76		3			
**d**	Signaling	SH3 domain-containing kinase-binding protein 1	2.7	comp52501	Q96B97	2.00E-08		1	4		
**d**	Signaling	SLIT-ROBO Rho GTPase-activating protein 3	14.5	comp55337	Q812A2	0		2			
**d**	Signaling	Sorbin and SH3 domain-containing protein 2	2.4	comp56783	O35413	3.00E-27		1			
**d**	Signaling	Uridine-cytidine kinase 2-B (EC:2.7.1.48)	2	comp50999	Q7ZV79	4.00E-157		4			
**d**	Signaling	von Willebrand factor D and EGF domain-containing protein	2.1	comp50126	Q8N2E2	2.00E-39		3			
**d**	Signaling, Stress response	Growth arrest and DNA damage-inducible protein GADD45 gamma	2	comp46772	Q9WTQ7	3.00E-61		1			
**d**	Stress response - molecular chaperone	Heat shock 70 kDa protein (Hsp70)	4	comp49050	Q91233	2.00E-14	X	3			
**d**	Stress response - molecular chaperone	Heat shock protein beta-11 (Hspb11)	3.1	comp46283	A5JV83	6.00E-76		2	6		
**d**	Stress response - molecular chaperone	Heat shock protein beta-7 (Hspb7)	3.1	comp29105	Q9UBY9	4.00E-60		1	3		
**d**	Stress response - oxidative stress	Eosinophil peroxidase (EC:1.11.1.7)	3.3	comp56586	P11678	0		2			
**d**	Stress response - oxidative stress	Mitochondrial uncoupling protein 2 (UCP2)	2.9	comp52795	P70406	1.00E-82	X	1			
**d**	Structural	Collagen alpha-1(I) chain	2.4	comp55856	P02457	1.00E-17		3			
**d**	Structural	Periplakin	3.1	comp47218	O60437	0		2	6		
**d**	Structural	Gliomedin	2.4	comp51177	Q6ZMI3	3.00E-59		6			
**d**	Structural - Cell adhesion	Collagen alpha-1(XI) chain	2.4	comp55502	P12107	0		3			
**d**	Structural - Cell adhesion	Collagen alpha-1(XVIII) chain	1.6	comp56713	P39061	2.00E-45		3			
**d**	Structural - Cell adhesion	Down syndrome cell adhesion molecule	2	comp52308	O60469	2.00E-49		3			

Heatmap Cluster	Category	UniProt Gene Name (Enzyme Code)	Max Fold Change	Trinity Contig	Uniprot Acc.	E-value	Both spp. ?	Significant Pairwise Comparison			
**d**	Structural - Cell adhesion	Transforming growth factor-beta-induced protein ig-h3	2	comp37127	Q15582	0		3			
**d**	Structural - Cytoskeletal	Keratin, type I cytoskeletal 17	3.3	comp42655	Q04695	3.00E-06		2	6		
**d**	Transcription	Aristaless-related homeobox protein	1.6	comp54697	O42115	4.00E-163		1			
**d**	Transcription	Four and a half LIM domains protein 1	2	comp56627	Q9WUH4	8.00E-131		1			
**d**	Transcription	Four and a half LIM domains protein 2	2.5	comp51429	Q14192	4.00E-172		1			
**d**	Transcription	Hypermethylated in cancer 2 protein	3.8	comp51956	Q90W33	5.00E-16	X	1	3	6	
**d**	Transcription	Occludin	3.1	comp53867	Q16625	2.00E-97		2	6		
**d**	Transcription	Thyroid hormone receptor-associated protein 3	2.7	comp54597	Q5M7V8	1.00E-45		1	6		
**d**	Transcription	Transcription factor LBX1	2.1	comp48136	Q2PYN8	8.00E-83		1			
**d**	Transcription	Transcriptional activator protein Pur-beta	1.9	comp53998	Q6PHK6	2.00E-61		3			
**d**	Translation	40S ribosomal protein S27-like	3.9	comp45956	Q3T0B7	2.00E-33		1	4	2	
**d**	Translation, post-translational modification	Probable polypeptide N-acetylgalactosaminyltransferase 8	3.1	comp47844	Q9NY28	0		2	6		
**d**	Transmembrane transport	ADP/ATP translocase 2	2.4	comp38041	P51881	1.00E-108		1			
**d**	Transport	Fatty acid-binding protein, heart	2.4	comp41908	O13008	2.00E-63		1			
**d**	Transport	Solute carrier family 35 member F5	2.6	comp51635	Q4R794	9.00E-61		1			
**e**	Actin cytoskeleton organization, structural	Actin-related protein 2/3 complex subunit 1A	2.7	comp14792	Q92747	7.00E-174		2			
**e**	Actin cytoskeleton organization, structural	Band 4.1-like protein 1	2	comp52914	Q9Z2H5	0		6			
**e**	Actin cytoskeleton organization, structural	FH2 domain-containing protein 1	1.6	comp46404	Q9C0D6	6.00E-86		2			
**e**	Bicarbonate transport	Carbonic anhydrase 6 (CA6) (EC:4.2.1.1)	2.9	comp14717	P23280	9.00E-99		2	6		
**e**	Calcium ion binding	Annexin A1	2.7	comp53489	P14087	5.00E-98		2			
**e**	Cell redox homeostasis	SH3 domain-binding glutamic acid-rich-like protein 3	4	comp14750	Q9H299	4.00E-24		6			
**e**	Cell-Cell adhesion	Cell surface glycoprotein MUC18	2.9	comp37784	Q8R2Y2	2.00E-08		2			
**e**	Cell-Cell adhesion	Claudin-8	3	comp49968	Q9Z260	3.00E-64		2			
**e**	Lipid transport	Oxysterol-binding protein-related protein 6	2.5	comp47289	Q8BXR9	0		2	6		
**e**	Metabolic process	Heparan-sulfate 6-O-sulfotransferase 1-A (EC:2.8.2)	2.4	comp54499	Q56UJ5	0		2			
**e**	Metabolism, lipid	Arachidonate 12-lipoxygenase, 12R-type	3.6	comp57271	O75342	4.00E-135		2	6		
**e**	Metabolism, lipid	HRAS-like suppressor 2	3.7	comp50565	Q9NWW9	2.00E-12		2	6		
**e**	Metabolism, lipid	Lipolysis-stimulated lipoprotein receptor	3.2	comp52838	Q86X29	1.00E-45		2			
**e**	NA	Cornifelin homolog B	4.1	comp46926	Q6DK99	7.00E-15		2	6		
**e**	NA	Neuroblast differentiation-associated protein AHNAK	4.1	comp57232	Q09666	2.00E-54		2	5	6	
**e**	NA	Transmembrane protein 54	2.7	comp47920	Q969K7	7.00E-14		6			
**e**	Nitrogen metabolism - glutamine biosynthesis	Glutamine synthetase (EC:6.3.1.2)	3.5	comp51758	P51121	0		2			
**e**	Plasma membrane repair	Myoferlin	2.8	comp52641	B3DLH6	0		2	4	6	
**e**	Proteolysis	CUB and zona pellucida-like domain-containing protein 1	5	comp51780	P70412	2.00E-25		2			
**e**	Proteolysis	Disintegrin and metalloproteinase domain-containing protein 9 (EC:3.4.24)	2.9	comp53157	Q61072	1.00E-66		2			
**e**	Proteolysis	Serine protease 27	2.8	comp49859	Q9BQR3	1.00E-64		2			
**e**	Proteolysis; Apoptosis	Apoptosis-associated speck-like protein containing a CARD	2.7	comp54601	Q9I9N6	2.00E-08		2	6		
**e**	Proteolysis; Apoptosis	Caspase-1 (EC:3.4.22)	2.7	comp56278	Q9MZV6	2.00E-06		6			

Heatmap Cluster	Category	UniProt Gene Name (Enzyme Code)	Max Fold Change	Trinity Contig	Uniprot Acc.	E-value	Both spp. ?	Significant Pairwise Comparison			
**e**	Proteolysis; Apoptosis	Caspase-1 (EC:3.4.22)	2.7	comp56797	Q9N2I1	9.00E-47		6			
**e**	RNA processing	Epithelial splicing regulatory protein 2	2.7	comp53471	Q7ZVR8	0		2	6		
**e**	Serine protease inhibitor	Kunitz-type protease inhibitor 1	3.5	comp54591	Q9R097	5.00E-99		2	6		
**e**	Signaling	Adenylate kinase 4, mitochondrial (EC:2.7.4.3; EC:2.7.4.10)	3.2	comp48783	Q5R421	2.00E-101		2	6		
**e**	Signaling	G-protein coupled receptor family C group 5 member C	2.4	comp52156	Q2YDG0	2.00E-23		6			
**e**	Signaling	Receptor-interacting serine/threonine-protein kinase 4 (EC:2.7.11)	2	comp46448	Q9ERK0	0		6			
**e**	Signaling	Serine/threonine-protein kinase Sgk1 (EC:2.7.11)	3.1	comp54007	Q5Q0U5	0		2	6		
**e**	Signaling	Tetraspanin-8	2.8	comp42593	Q8R3G9	4.00E-34		6			
**e**	Stress response - oxidative stress	Cell surface superoxide dismutase [Cu-Zn] 5	3.3	comp53894	Q5AD07	2.00E-07		2	6		
**e**	Structural	Chitin synthase 8	2	comp51611	Q4P9K9	7.00E-20		2			
**e**	Structural	Envoplakin	2.1	comp45514	Q92817	0		2	6		
**e**	Structural	Sciellin	3.5	comp52432	O95171	7.00E-24		6			
**e**	Structural - Cell adhesion	Junctional adhesion molecule A	2.8	comp51387	O88792	1.00E-47		2	6		
**e**	Structural - Cytoskeletal	Keratin, type II cytoskeletal 8	4.4	comp14773	Q6NWF6	1.00E-13		2			
**e**	Transcription	Grainyhead-like protein 1 homolog	3.5	comp57108	Q9NZI5	0		2	6		
**e**	Transcription	Grainyhead-like protein 2 homolog	2.8	comp56907	Q5M7R9	2.00E-95		2	6		
**e**	Transcription	Grainyhead-like protein 3 homolog	2.5	comp48898	Q6GL65	2.00E-146		2	6		
**e**	Transcription	Krueppel-like factor 2	3.6	comp52076	Q60843	5.00E-58	X	2	6		
**e**	Transcription	Nuclear receptor subfamily 1 group D member 2	2.4	comp54949	Q14995	5.00E-90	X	1	2		
**e**	Transcription	RBBP8 N-terminal-like protein (EC:3.1)	1.9	comp54677	Q8NC74	9.00E-31		2	6		
**e**	Transcription	Serum deprivation-response protein	4.5	comp42549	O95810	3.00E-83		2			
**e**	Transcription	Tripartite motif-containing protein 16	3.8	comp48965	Q99PP9	2.00E-66		2			
**e**	Transcription	Tumor protein 63	3.7	comp51153	O88898	0		2			
**e**	Transcription	Zinc finger protein RFP	4.3	comp56403	P14373	2.00E-59		2			
**e**	Transcription, Stress response	CCAAT/enhancer-binding protein delta (C/EBPD)	1.8	comp41824	P49716	3.00E-53	X	1	2		
**e**	Translation	Epithelial splicing regulatory protein 1	2.6	comp53316	Q6NXG1	0		2	6		
**e**	Transport	Synaptotagmin-like protein 2	2	comp54026	Q99N50	7.00E-121		6			
**e**	Transport, endocytosis	Limb region 1 homolog-like protein	2.4	comp54240	Q803C7	0		6			
**f**	Carbohydrate metabolism - glycolysis	Glyceraldehyde-3-phosphate dehydrogenase 2 (EC:1.2.1.12)	2.6	comp30238	Q5MJ86	0		2			
**f**	Cell cycle	Cell cycle control protein 50B	3.2	comp52086	Q8BHG3	9.00E-99		2	6		
**f**	Cell-Cell adhesion	Desmoglein-2	2.2	comp55925	Q14126	9.00E-110		2			
**f**	Cell-Cell adhesion	Desmoglein-2	10.3	comp46106	O55111	4.00E-77		2			
**f**	Cell-substrate adhesion	Alpha-tectorin	2.8	comp57361	O08523	1.00E-80		2			
**f**	Immune	Carcinoembryonic antigen-related cell adhesion molecule 5	5.2	comp54442	P06731	6.00E-28		2	6		
**f**	Immune	Chitotriosidase-1	5.8	comp56218	Q13231	3.00E-08		2	6		
**f**	Lipid transport	Extended synaptotagmin-3	2.4	comp56832	Q5M7N9	2.00E-51		2	6		
**f**	Lipid transport	Probable phospholipid-transporting ATPase IC (EC:3.6.3.1)	2.5	comp56357	Q5BL50	0		2	6		
**f**	Metabolic process	GTPase IMAP family member 7	2.6	comp50360	Q8NHV1	2.00E-27		2			
**f**	Metal ion binding	Zinc finger protein 185	2.6	comp53443	O15231	6.00E-15		6			
**f**	Motor activity	Cingulin	2.4	comp54556	A9X1A5	2.00E-15		2			
**f**	NA	Uncharacterized protein KIAA1522 homolog	3	comp54780	Q1LWM5	5.00E-26		2			
**f**	NA	Uncharacterized protein KIAA1522 homolog	2.5	comp52102	Q1LWM5	3.00E-09		2			
**f**	Pathogenesis	Galactose-specific lectin nattectin	12	comp37307	Q66S03	2.00E-36		2	6		

Heatmap Cluster	Category	UniProt Gene Name (Enzyme Code)	Max Fold Change	Trinity Contig	Uniprot Acc.	E-value	Both spp. ?	Significant Pairwise Comparison			
**f**	Pathogenesis	Galactose-specific lectin nattectin	10.1	comp52980	Q66S03	1.00E-18		2	6		
**f**	Proteolysis	Calpain small subunit 1	2.3	comp44563	P04632	1.00E-98		2			
**f**	Proteolysis	Stromelysin-3 (EC:3.4.24)	2.4	comp54398	Q11005	1.00E-164		2			
**f**	Proteolysis	Suppressor of tumorigenicity 14 protein homolog (EC:3.4.21)	2.8	comp51884	P56677	0		2	6		
**f**	Proteolysis	Suppressor of tumorigenicity 14 protein homolog (EC:3.4.21)	3.1	comp48613	P56677	0		2			
**f**	Proteolysis	Transmembrane protease serine 4 (EC:3.4.21)	2.3	comp54302	Q9NRS4	1.00E-87		2	6		
**f**	Proteolysis, endopeptidase	Calpain-9	3.5	comp53570	O35920	6.00E-150		2	6		
**f**	Signaling	Protein S100-A16	2.7	comp48109	Q0VCM0	9.00E-10		2	6		
**f**	Signaling	Receptor-type tyrosine-protein phosphatase zeta (EC:3.1.3.48)	2.3	comp55823	P23471	0		2	6		
**f**	Signaling	Rho guanine nucleotide exchange factor 5	2.4	comp55203	Q12774	3.00E-134		6			
**f**	Signaling, Stress response	14-3-3 protein beta/alpha-1	2.7	comp47075	Q6UFZ9	1.00E-121		2	6		
**f**	Stress response, protein deacetylation	NAD-dependent protein deacylase sirtuin-5, mitochondrial (EC:3.5.1)	3.5	comp49791	F7DKV7	6.00E-149		2			
**f**	Structural	Envoplakin	3	comp54331	Q92817	8.00E-145		2	6		
**f**	Structural - Cell adhesion	Plakophilin-1	3.5	comp53905	P97350	3.00E-127		2			
**f**	Structural - Cell adhesion	Plakophilin-3	3.4	comp55362	Q08DQ0	2.00E-143		2	4	6	
**f**	Transcription	Homeobox protein Dlx3b	2.6	comp47120	Q01702	3.00E-144		6			
**f**	Translation	Clustered mitochondria protein homolog	1.8	comp55347	Q5SW19	0		2			
**g**	Actin binding	Desmoplakin	4.9	comp57051	E9Q557	1.0E-104		2	4		
**g**	Actin binding	Destrin	5	comp29410	P18359	7.0E-20		6			
**g**	Actin binding	Protein cordon-bleu	2	comp56159	E7F568	1.0E-37		3			
**g**	Actin binding	Adseverin	4.3	comp47755	Q5ZIV9	2.0E-42		2	4	6	
**g**	Actin bundling protein	Plastin-3	8.2	comp45193	O88818	0		2	3	4	6
**g**	Calcium ion homeostasis	C-C motif chemokine 28	5.4	comp37958	Q9NRJ3	1.00E-05		2	6		
**g**	Cell differentiation	Chordin-like protein 2	2.5	comp45546	Q6WN34	3.00E-114		4			
**g**	Cell-Cell adhesion	Cadherin-like protein 26	4.2	comp51425	Q8IXH8	1.00E-17		2	6		
**g**	Cell-Cell adhesion	Claudin-4	3.9	comp50450	Q6BBL6	4.00E-75		2	6		
**g**	Cell-Cell adhesion	Claudin-7-B	6	comp44670	Q9YH92	3.00E-77		2	6		
**g**	Cell-Cell adhesion	Desmoglein-2	4.7	comp50714	Q14126	1.00E-50		2	4	6	
**g**	Ion transport	Conoporin-Cn1	4.1	comp37571	P0DKQ8	9.00E-08		2	4		
**g**	Ion transport	Cytolysin Src-1	4.2	comp44246	Q86FQ0	5.00E-13		2	4		
**g**	Ion transport	Cytolysin-3	4.5	comp34136	Q9U6X1	2.00E-09		2	6		
**g**	Lipid transport	Apolipoprotein Eb	4.5	comp45944	O42364	1.00E-106		2			
**g**	Metabolism, lipid	Inositol monophosphatase 1 (EC:3.1.3.25)	3.6	comp14781	P29218	4.00E-120		2	4		
**g**	Metabolism, lipid	Lipocalin (EC:5.3.99.2)	4.7	comp41807	Q01584	9.00E-19		2			
**g**	Metabolism, lipid	Lipocalin (EC:5.3.99.2)	2.5	comp41876	Q01584	8.00E-30		1	4		
**g**	NA	Epiplakin	5.4	comp52507	P58107	2.00E-103		2			
**g**	NA	Fin bud initiation factor	2.2	comp47492	A1IGX5	3.00E-78		4			
**g**	NA	Keratocan	2.6	comp45519	Q9DE66	2.00E-122		4			
**g**	Signaling	Agouti-related protein	6.9	comp44826	P56413	7.00E-08		2	3	4	6
**g**	Signaling	Agouti-signaling protein	4.2	comp43528	Q03288	2.00E-08		3			
**g**	Signaling	Fibrinogen-like protein 1	2.7	comp45926	Q3SZZ7	9.00E-70		2	4		
**g**	Signaling	Tumor-associated calcium signal transducer 2	4.4	comp37561	Q8BGV3	3.00E-52		2	6		
**g**	Stress response - molecular chaperone	Peptidyl-prolyl cis-trans isomerase (EC:5.2.1.8)	3.8	comp37028	P25007	2.00E-71		2	6		

Heatmap Cluster	Category	UniProt Gene Name (Enzyme Code)	Max Fold Change	Trinity Contig	Uniprot Acc.	E-value	Both spp. ?	Significant Pairwise Comparison			
**g**	Stress response - oxidative stress	Thioredoxin	3.3	comp51397	Q9DGI3	2.00E-28		6			
**g**	Structural	Epithelial membrane protein 2	3.9	comp46820	A5A6N6	2.00E-33		2	3	4	6
**g**	Structural - Cell adhesion	Cell surface A33 antigen	5.3	comp44957	Q99795	3.00E-41		2			
**g**	Structural - Cytoskeletal	Keratin, type II cytoskeletal 8	4.9	comp42144	P05787	3.00E-18		2	4		
**g**	Translation	Eukaryotic translation initiation factor 4E type 3	3.3	comp49705	Q66HY7	2.00E-113		3			
**g**	Transport	Retinol-binding protein 4-A	1.9	comp29267	P24774	4.00E-101		1			
**g**	Water transport	Aquaporin-3	3.6	comp51504	Q8R2N1	6.00E-126		2			
