# Supplementary material for: Species-Specific Responses of Juvenile Rockfish to Elevated pCO2: From Behavior to Genomics
Source: PLoS One. 2017 Jan 5;12(1):e0169670. doi: 10.1371/journal.pone.0169670 (PMC5215853; doi:10.1371/journal.pone.0169670)
Supplement: S4 Table — (PDF) [file pone.0169670.s004.pdf]

**S4 Table.** Gene Ontology (GO) consortium accession numbers from Fig 2B in the main text.

| **BIOLOGICAL PROCESS** | **GO Accession** |
|---|---|
| Apoptotic process | GO:0006915 |
| Biological adhesion | GO:0022610 |
| Biological regulation | GO:0065007 |
| Cellular component organization or biogenesis | GO:0071840 |
| Cellular process | GO:0009987 |
| Developmental process | GO:0032502 |
| Immune system process | GO:0002376 |
| Localization | GO:0051179 |
| Metabolic process | GO:0008152 |
| Multicellular organismal process | GO:0032501 |
| Reproduction | GO:0000003 |
| Response to stimulus | GO:0050896 |
| **MOLECULAR FUNCTION** | **GO Accession** |
| Antioxidant activity | GO:0016209 |
| Binding | GO:0005488 |
| Catalytic activity | GO:0003824 |
| Enzyme regulator activity | GO:0030234 |
| Nucleic acid binding transcription factor activity | GO:0001071 |
| Protein binding transcription factor activity | GO:0000988 |
| Receptor activity | GO:0004872 |
| Structural molecule activity | GO:0005198 |
| Translation regulator activity | GO:0045182 |
| Transporter activity | GO:0005215 |
