# Supplementary material for: Species-Specific Responses of Juvenile Rockfish to Elevated pCO2: From Behavior to Genomics
Source: PLoS One. 2017 Jan 5;12(1):e0169670. doi: 10.1371/journal.pone.0169670 (PMC5215853; doi:10.1371/journal.pone.0169670)

S1 Fig. pH time series (A) from Carmel Bay, California collected using a SeaFET sensor (with a Durafet ISFET pH electrode) deployed to the benthos in the center of a large kelp bed at 12 m depth (B). pH readings were made every 15 minutes and are plotted as hourly means. The gap from May to October 2013 occurred due to a flooded housing.

A.

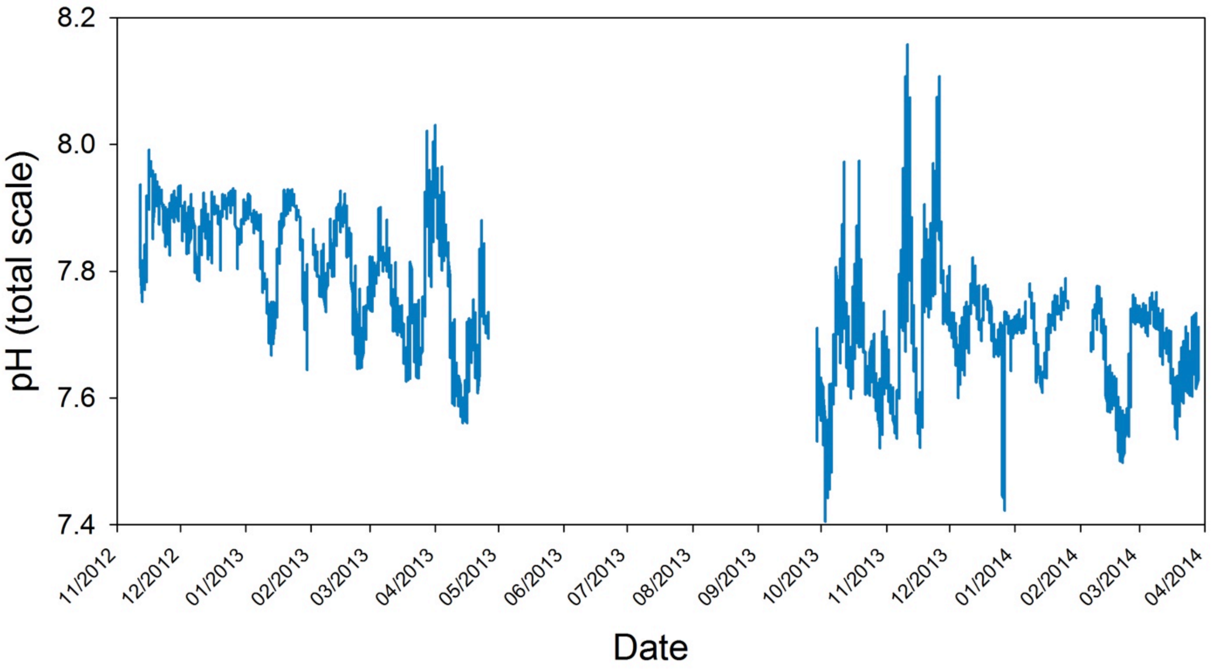

B.

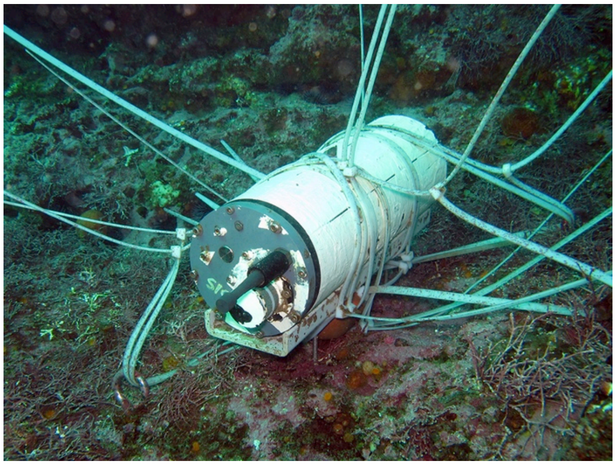

Supplement: S1 Fig — pH readings were made every 15 minutes and are plotted as hourly means. The gap from May to October 2013 occurred due to a flooded housing. (PDF) [file pone.0169670.s005.pdf]
