# Supplementary material for: Species-Specific Responses of Juvenile Rockfish to Elevated pCO2: From Behavior to Genomics
Source: PLoS One. 2017 Jan 5;12(1):e0169670. doi: 10.1371/journal.pone.0169670 (PMC5215853; doi:10.1371/journal.pone.0169670)

S3 Fig. Growth rates (mm d^{-1}) measured as a change in length over the experimental exposure duration for copper and blue rockfish as a function of pCO_2 treatment history. Bars are mean values (± SE). Letters over bars represent results of Tukey HSD post-hoc tests; significantly different means do not share letters in common.

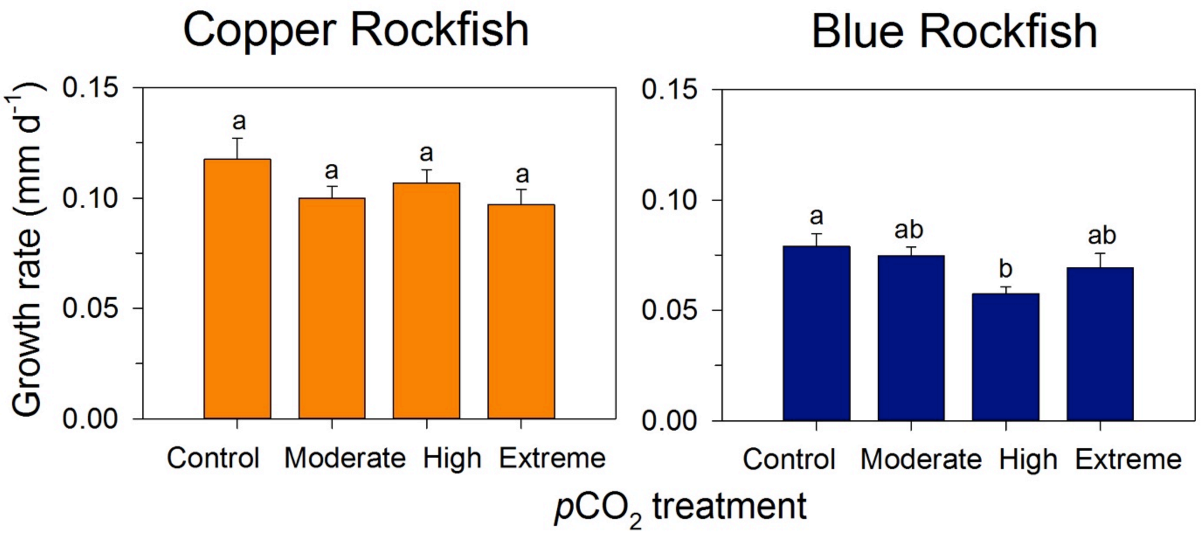

Supplement: S3 Fig — Bars are mean values (± SE). Letters over bars represent results of Tukey HSD post-hoc tests; significantly different means do not share letters in common. (PDF) [file pone.0169670.s007.pdf]
