# Supplementary material for: Species-Specific Responses of Juvenile Rockfish to Elevated pCO2: From Behavior to Genomics
Source: PLoS One. 2017 Jan 5;12(1):e0169670. doi: 10.1371/journal.pone.0169670 (PMC5215853; doi:10.1371/journal.pone.0169670)

S4 Fig. Routine metabolic rate and maximum metabolic rate of copper and blue rockfish. (A,B) Routine metabolic rate was measured as the oxygen consumption rate while at rest. (C,D) Maximum metabolic rate was measured as the oxygen consumption rate calculated while the fish swam at its presumed maximum rate in a swim tunnel. Letters over bars represent results of Tukey HSD post-hoc tests; significantly different means do not share letters in common.

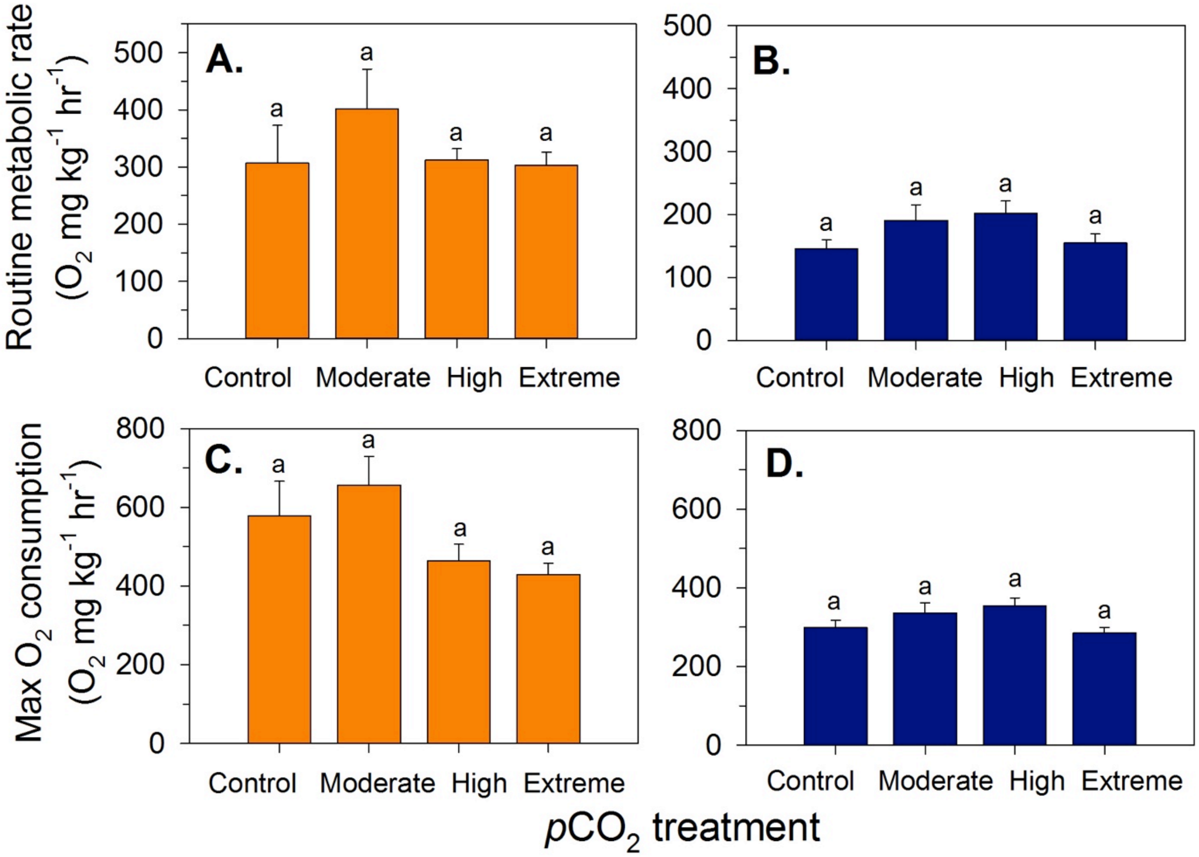

Supplement: S4 Fig — (A,B) Routine metabolic rate was measured as the oxygen consumption rate while at rest. (C,D) Maximum metabolic rate was measured as the oxygen consumption rate calculated while the fish swam at its presumed maximum rate in a swim tunnel. Letters over bars represent results of Tukey HSD post-hoc tests; significantly different means do not share letters in common. (PDF) [file pone.0169670.s008.pdf]
