# Supplementary material for: Species-Specific Responses of Juvenile Rockfish to Elevated pCO2: From Behavior to Genomics
Source: PLoS One. 2017 Jan 5;12(1):e0169670. doi: 10.1371/journal.pone.0169670 (PMC5215853; doi:10.1371/journal.pone.0169670)

S5 Fig. Principal component analysis using an unsupervised analysis of all gene transcripts. Treatment groupings are similar to those observed in the heatmaps for differentially expressed gene patterns (Fig. 3). A) For copper rockfish, PC2 (12.9%) and PC3 (11.6%) separated pCO_2 treatments into the two highest and lowest pCO_2 treatments. The variance explained by PC1 (13.8%) did not differ greatly from PC2 and PC3. B) For blue rockfish, PC1 (18.3%) and PC2 (13.8%) separated the control and extreme pCO_2 treatments and clustered the moderate treatments.

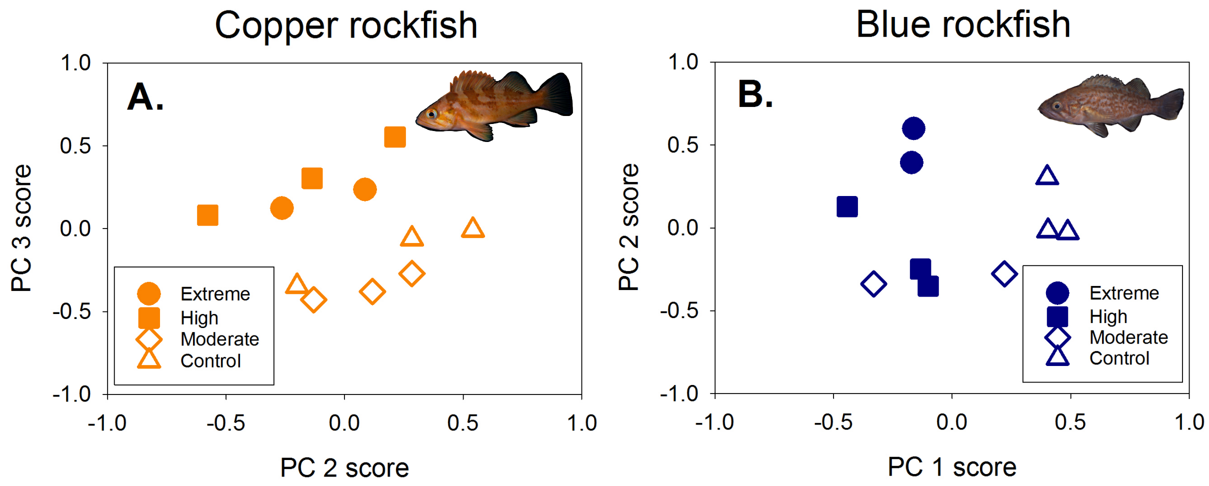

Supplement: S5 Fig — Treatment groupings are similar to those observed in the heatmaps for differentially expressed gene patterns (Fig 3). A) For copper rockfish, PC2 (12.9%) and PC3 (11.6%) separated pCO2 treatments into the two highest and lowest pCO2 treatments. The variance explained by PC1 (13.8%) did not differ greatly from PC2 and PC3. B) For blue rockfish, PC1 (18.3%) and PC2 (13.8%) separated the control and extreme pCO2 treatments and clustered the moderate treatments. (PDF) [file pone.0169670.s009.pdf]
