# Supplementary material for: Species-Specific Responses of Juvenile Rockfish to Elevated pCO2: From Behavior to Genomics
Source: PLoS One. 2017 Jan 5;12(1):e0169670. doi: 10.1371/journal.pone.0169670 (PMC5215853; doi:10.1371/journal.pone.0169670)

S6 Fig. Molecular function and biological process Gene Ontology (GO) categories represented in the annotated copper rockfish *de novo* transcriptome assembly. Compared to the full assembly, differentially expressed genes (Fig 3B) showed increased expression of genes involved in transcription activity and biological regulation in copper rockfish and increased expression of genes involved in structural molecule activity in blue rockfish.

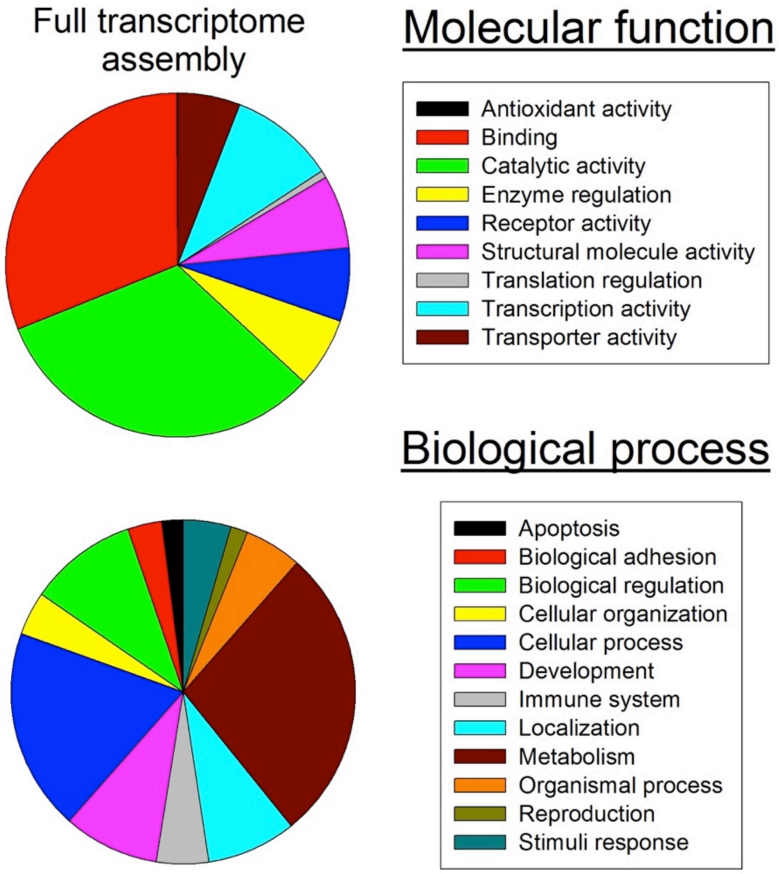

Supplement: S6 Fig — Compared to the full assembly, differentially expressed genes (Fig 3B) showed increased expression of genes involved in transcription activity and biological regulation in copper rockfish and increased expression of genes involved in structural molecule activity in blue rockfish. (PDF) [file pone.0169670.s010.pdf]
